# Eﬄux in the Oral Metagenome: The Discovery of a Novel Tetracycline and Tigecycline ABC Transporter

**DOI:** 10.3389/fmicb.2016.01923

**Published:** 2016-12-06

**Authors:** Liam J. Reynolds, Adam P. Roberts, Muna F. Anjum

**Affiliations:** ^1^Department of Microbial Diseases, UCL Eastman Dental Institute, Faculty of Medical Sciences, University College LondonLondon, UK; ^2^Department of Bacteriology, Animal and Plant Health AgencyAddlestone, UK

**Keywords:** tetracycline, tigecycline, metagenomics, ABC transporter, fitness, antibiotic resistance

## Abstract

Antibiotic resistance in human bacterial pathogens and commensals is threatening our ability to treat infections and conduct common medical procedures. As novel antibiotics are discovered and marketed it is important that we understand how resistance to them may arise and know what environments may act as reservoirs for such resistance genes. In this study a tetracycline and tigecycline resistant clone was identified by screening a human saliva metagenomic library in *Escherichia coli* EPI300 on agar containing 5 μg/ml tetracycline. Sequencing of the DNA insert present within the tetracycline resistant clone revealed it to contain a 7,765 bp fragment harboring novel ABC half transporter genes, *tet*AB(60). Mutagenesis studies performed on these genes confirmed that they were responsible for the tetracycline and tigecycline resistance phenotypes. Growth studies performed using *E. coli* EPI300 clones that harbored either the wild type, the mutated, or none of these genes indicated that there was a fitness cost associated with presence of these genes, with the isolate harboring both genes exhibiting a significantly slower growth than control strains. Given the emergence of *E. coli* strains that are sensitive only to tigecycline and doxycycline it is concerning that such a resistance mechanism has been identified in the human oral cavity.

## Introduction

Tetracyclines are a group of broad spectrum antibiotics that have recently faced a reduction in clinical use due to the increase in prevalence of tetracycline resistance (Chopra and Roberts, 2001; Bishburg and Bishburg, 2009). In the UK tetracyclines are the most sold antibiotic for animal use and represent 10% of prescribed antibiotics for human clinical use. This widespread use of tetracyclines exerts a selection pressure on microorganisms to maintain tetracycline resistance genes (Roberts, 2003; Martinez, 2009; Wu et al., 2010). Resistance is mainly attributed to the production of eﬄux pumps, ribosomal protection proteins (RPPs) that prevent tetracycline binding to the ribosome, and less often, tetracycline degrading enzymes (Aminov et al., 2002; Connell et al., 2003; Yang et al., 2004; Forsberg et al., 2015).

Although resistance to this group of antibiotics is prevalent they are still used in the treatment of some human infections, including *Chlamydia* infections and some eye infections such as trachoma (Hu et al., 2010; Dukers-Muijrers et al., 2015). Tigecycline is a novel semi-synthetic derivative of tetracycline and the first of the glycylcyclines. It contains a bulky N,N-dimethylglycylamido side group that allows it to overcome RPP and eﬄux mechanisms of resistance to earlier generation precursors such as tetracycline (Someya et al., 1995; Olson et al., 2006). Tigecycline is used in the treatment of skin and abdominal infections as well as some cases of community acquired pneumonia (Rubinstein and Vaughan, 2005; Shen et al., 2015; Van Berkel et al., 2016). It has been shown that tetracycline resistance genes can obtain mutations that broaden the activity of their products to new tetracycline derivatives (Linkevicius et al., 2015). It is important that we understand the mechanisms of resistance to our current generation of tetracyclines in order for us to identify environments that may harbor genes that could confer resistance to novel tetracyclines, including those that are still in development.

The microbiota of the human oral cavity constitutes a reservoir of tetracycline resistance genes. RPP genes such as *tet*(M) are the most abundant tetracycline resistant genes in bacteria of the oral cavity (Villedieu et al., 2003; Seville et al., 2009). Tetracycline eﬄux genes such as major facilitator superfamily (MFS) exporters including *tet*(L) and the ATP Binding Cassette (ABC) transporter *tet*AB(46) have also been detected in bacteria in the oral cavity (Lancaster et al., 2005; Seville et al., 2009; Warburton et al., 2013).

ABC transporters are a functionally and structurally diverse family of proteins. They may comprise a single peptide, four peptides or two half transporters. Bacterial half ABC transporters typically contain a transmembrane domain (TMD) and a highly conserved nucleotide binding domain (NBD). Functional dimeric ABC transporters are composed of two half ABC transporter subunits each contributing a TMD spanning the membrane to form a substrate channel (Biemans-Oldehinkel et al., 2006; Dawson and Locher, 2006). The two NBDs interact to form the ABC of the transporter at the cytoplasmic face of the membrane that can bind two ATP molecules (Jones and George, 1999). It is here that ATP binding and hydrolysis triggers conformational changes in the substrate channel (Hollenstein et al., 2007). Cycles of ATP binding and hydrolysis allow the substrate channel to alternate between being open to the cytoplasm for substrate binding and open to the cells external environment or periplasm for substrate eﬄux (Higgins, 2001; Hellmich et al., 2012). ABC transporters that confer multidrug resistance (MDR) and biocide resistance to bacteria including human pathogens have been described such as YheH/I, LmrCD, PatAB and EfrAB from *Bacillus subtilis, Lactococcus lactis, Streptococcus pneumoniae* and *Enterococcus faecalis*, respectively (Lee et al., 2003; Lubelski et al., 2004; Torres et al., 2009; Baylay et al., 2015).

The aim of this study was to identify the gene(s) conferring resistance in a tetracycline and tigecycline resistant clone that was identified from a human saliva metagenomic library in *Escherichia coli*. Two genes from the clone, *tet*A(60) and *tet*B(60) were found to encode for two half transporter proteins which were shown to be responsible for the observed antibiotic resistance phenotype and reduced fitness.

## Materials and Methods

### Strains and Culture Conditions

The strains used in this study are listed in **Table 1**. *E. coli* EPI300 strains were cultured in Luria-Bertani broth (LB; Sigma-Aldrich^®^) and LB agar (LA; Life Technologies^TM^) at 37°C with shaking at 200 rpm for liquid culture. When antibiotic selection was required the media was supplemented with chloramphenicol (12.5 μg/ml; Sigma-Aldrich^®^) and tetracycline (5 μg/ml; Sigma-Aldrich^®^). Mueller Hinton (MH; Sigma-Aldrich^®^) agar was used in disk diffusion assays.

**Table 1:** Bacterial strains, plasmids and constructs used in this study.

	Name	Information	Source
Vectors	pCC1BAC	Chloramphenicol resistance marker, inducible to multicopy in *E. coli* EPi300	Epicentre^®^ CopyControl^TM^
	pHSG396	Chloramphenicol resistance marker	Takara Bio^©^
Constructs	pCC1BAC::PS9	pCC1BAC containing 7,765 bp metagenomic DNA insert	This study
	pHSG396::*tet*A(60)	pHSG396::*tet*A(60)	This study
	pHSG396::*tet*B(60)	pHSG396::*tet*B(60)	This study
	pHSG396::*tet*AB(60)	pHSG396::*tet*AB(60)	This study
	pHSG396::*tet*B(60)Δ*tet*A(60)	pHSG396::*tet*B(60)Δ*tet*A(60)	This study
	pHSG396::*tet*A(60)Δ*tet*B(60)	pHSG396::*tet*A(60)Δ*tet*B(60)	This study
Bacterial Strains	*E. coli* EPI300	Electrocompetent, inducible trfA gene for pCC1BAC copy number control	Epicentre^®^ CopyControl^TM^
	*E. coli*::pCC1BAC	*E. coli* EPI300::pCC1BAC	P. Warburton, Eastman Dental Institute
	*E. coli*::pHSG396	*E. coli* EPI300::pHSG396	This study
	PS9	*E. coli* EPI300::[pCC1BAC::PS9]	This study
	*E. coli*::pHSG396*tet*A(60)	*E. coli* EPI300::[pHSG396::*tet*A(60)]	This study
	*E. coli*::pHSG396*tet*B(60)	*E. coli* EPI300::[pHSG396::*tet*B(60)]	This study
	*E. coli*::pHSG396*tet*AB(60)	*E. coli* EPI300::[pHSG396::tetAB(60)]	This study
	*E. coli*::pHSG396*tet*B(60)Δ*tet*A(60)	*E. coli* EPI300::[pHSG396::*tet*B(60)Δ*tet*A(60)]	This study
	*E. coli*::pHSG396*tet*A(60)Δ*tet*B(60)	*E. coli* EPI300::[pHSG396::*tet*A(60)Δ*tet*B(60)]	This study

### Sample Collection and Metagenomic DNA Extraction

Saliva samples were collected from 11 healthy individuals who had not taken antibiotics within the previous 3 months. Saliva was expectorated into sterile tubes (approximately 5 ml per individual) and samples were pooled. Metagenomic DNA was extracted in 1.5 ml aliquots using a modified protocol of the Gentra Puregene Yeast/Bact. Kit (Qiagen) as previously described (Seville et al., 2009). Ethical approval to collect human saliva from volunteers was granted by the UCL Research Ethics Committee (Project ID Number 5017/001).

### Creation of a Metagenomic Library

Saliva metagenomic DNA was partially digested using HindIII, ligated into pCC1BAC and transformed into *E. coli* EPI300 as described previously (Seville et al., 2009).

After transformation, cells were recovered in SOC media (New England Biolabs^®^), cultured on LA containing chloramphenicol, 0.1 mM isopropyl β-D-1-thiogalactopyranoside (IPTG; Promega^©^) and 40 μg/ml 5-bromo-4-chloro-3-indolyl-β-D-galactopyranoside (X-gal; Promega^©^) for 16 h. White clones were cultured in LB with chloramphenicol in individual wells of 96-well plates at 37°C for 16 h. The cultures were then stored at -80°C in 20% glycerol.

### Screening of Metagenomic Library and Resistant Clone Isolation

Approximately 27,000 clones of the metagenomic library were screened for tetracycline resistance by plating the library onto LA with chloramphenicol (12.5 μg/ml) and tetracycline (5 μg/ml) and incubating them at 37°C for 16 h. A tetracycline resistant clone, PS9, was selected for further study.

### DNA Sequencing, Analysis and Annotation

A list of the primers used in this study is detailed in Supplementary Table S1. Sequencing of the BAC clone in PS9 was accomplished using 454 sequencing as described previously (Card et al., 2014). Sequencing of subclones and mutants was conducted using primer extension Sanger sequencing by Beckman Coulter Genomics Inc. Contigs were assembled using SeqMan Pro (Lasergene software, DNASTAR, Madison, WI, USA) and sequence gaps were closed using PCR and Sanger sequencing (Sanger et al., 1977). Sequences were analyzed using the tools on NCBI. Two open reading frames (ORFs) encoding hypothetical ABC half transporter genes were named *tet*A(60) and *tet*B(60) by the Stuart B. Levy lab according to tetracycline resistance gene nomenclature guidelines (Levy et al., 1999). The sequences for tetA(60) and tetB(60) were submitted to GenBank (accession numbers KX000272.1and KX000273.1). The full 7,765 bp insert sequence was also submitted to Genbank (accession number KX887332; Supplementary Figure S1C). The putative amino acid sequences of TetA(60) and TetB(60) were compared to other phenotypically validated tetracycline and multidrug ABC transporter protein sequences from Gram-positive bacteria [TetAB(60), YheH/I, LmrCD, PatAB and EfrAB] by alignment using Clustal Omega at http://www.ebi.ac.uk/Tools/msa/clustalo/.

### Subcloning

Primers used for subcloning are detailed in Supplementary Table S1. Regions of PS9 were amplified using primer pairs that introduced flanking HindIII and BamHI sites. The amplified fragments were ligated into pHSG396 and transformed into *E. coli* EPI300.

### Mutagenesis

Primers used for mutagenesis are listed in Supplementary Table S1. In frame deletions of the Walker-A motifs of *tet*A(60) and *tet*B(60) were made using the NEB Q5 Site Directed Mutagenesis kit. Two pairs of non-overlapping primers were designed to amplify the pHSG396 vector containing both transporter genes. The first primer pair amplified pHSG396::*tet*AB(60) without a 69 bp region containing the *tet*A(60) Walker-A motif, keeping *tet*B(60) full length. The second primer pair amplified pHSG396::*tet*AB(60) without a 57 bp region containing the Walker-A motif of *tet*B(60) keeping *tet*A(60) intact. The resulting PCR products were circularized and transformed into *E. coli* EPI300.

### Disk Diffusion Assays

The susceptibilities of *E. coli* EPI300, *E. coli*::pHSG396 and *E. coli*::pHSG396tetAB(60) to various antibiotics (cefotaxime, ceftazidime metronidazole, neomycin, ciprofloxacin, nalidixic acid, gentamicin, amikacin, amoxicillin/clavulanate and trimetoprim/sulfametoxazole and erythromycin) were evaluated using the disk diffusion assay according to BSAC guidelines (Andrews, 2001). The antibiotic disks and concentrations used in this study are listed in **Table 2**.

**Table 2:** List of antibiotic disks used.

Antibiotic (Concentration)	Concentration
Cefotaxime	30 μg
Metronidazole	50 μg
Neomycin	10 μg
Ciprofloxacin	1 μg
Nalidixic acid	30 μg
Gentamicin	10 μg
Amoxicillin/Clavulanate	20 μg/10 μg
Trimetoprim/Sulfametoxazole	23.75 μg/1.25 μg
Amikacin	30 μg
Tetracycline	10 μg
Ceftazidime	30 μg
Erythromycin	5 μg

### Minimum Inhibitory Concentration (MIC) Assays

Minimum Inhibitory Concentrations of tetracycline, minocycline, and tigecycline were determined using the microbroth dilution method according to European Committee on Antimicrobial Susceptibility Testing (EUCAST) guidelines (EUCAST, 2015). For MIC determination, overnight cultures grown in LB were adjusted to an OD_600_ of 0.1; 10 μl of the adjusted overnight cultures were used to inoculate 90 μl fresh LB containing varying concentrations of tetracycline (0.25–32 μg/ml), minocycline (0.25–10 μg/ml) or tigecycline (0.25–10 μg/ml) in a 96 well plate format. These plates were incubated overnight at 37°C with shaking at 200 rpm. Growth was determined by spectrophotometry at OD_600_ and the MIC was determined as the lowest concentration of antibiotic that inhibited growth.

### Growth Curves

Overnight cultures of *E. coli*::pHSG396, *E. coli*::pHSG396*tet*AB(60), *E. coli*::pHSG396*tet*B(60)Δ*tet*A(60) and *E. coli*::pHSG396*tet*A(60)Δ*tet*B(60) grown in LB with chloramphenicol (12.5 μg/ml) and tetracycline (5 μg/ml; when required) were adjusted to an OD_600_ of 0.05 in LB and chloramphenicol (12.5 μg/ml). Cell suspensions were grown at 37°C with shaking at 200 rpm for 7 h and their cell density was measured every 30 min using spectrophotometry (OD_600_). *E. coli*::pHSG396*tet*AB(60)was also grown in LB and chloramphenicol (12.5 μg/ml) with tetracycline (5 μg/ml) to determine if the presence of this antibiotic affected the clones. Growth rates were measured for each clone as the slope of the line between two time points on the growth curve. The equation N_t_ = N_0_^∗^(1 + r)^t^, was used to calculate the maximum growth rate between 60 and 240 min. Technical triplicates and biological triplicates were conducted for all growth curves and growth rate calculations.

### Statistical Analysis

Standard deviations were calculated for each clone using the data obtained from the growth curve assays, which included nine data points encompassing biological and technical replicates. Standard deviations were used as error bars in **Figure 2** for comparison of the mean OD_600_ for each clone. Two tailed *t*-tests with 95% confidence intervals were used to determine the significance of differences between clones and the control (*E. coli*::pHSG396) in terms of OD_600_ at 420 min and growth kinetics.

## Results

A library of 27,000 clones was constructed from the pooled human saliva of 11 individuals. Screening of this metagenomic library for tetracycline resistant clones resulted in the isolation of two of clones capable of growing on tetracycline (5 μg/ml), including PS9.

**FIGURE 1:**
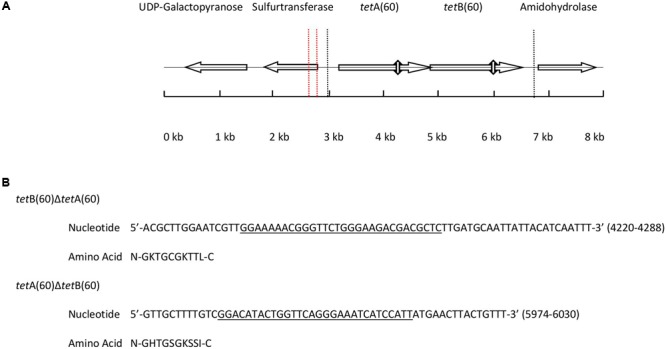
**(A)** Diagram depicting the position and orientation of the ORFs present in the 7,765 base pair insert of PS9 according to BlastX. The 3, 703 base pair region containing *tet*AB(60) that were subcloned to create pHSG396::*tet*AB(60) is marked by dashed lines. The positions of the Walker A motifs that were deleted to make pHSG396::*tet*B(60)Δ*tet*A(60) and pHSG396::*tet*A(60)Δ*tet*B(60) are marked by vertical double headed arrows and the inversion in the sequence is indicated by vertical dashed red lines. **(B)** The nucleotide sequences of the deleted regions are given above with the Walker A motif of each gene underlined and translated.

Analysis of the 7,765 bp PS9 insert revealed it to have nucleotide similarity along its entire length with *Streptococcus* sp. 263_SSPC (accession: GCA_001071995.1, 98% cover and 90% identity) and *Granulicatella adiacens* ATCC 49175 (accession: NZ_ACKZ00000000, 94% cover and 92% identity). The alignments also identified an inversion in the PS9 insert between 1,600 bp and 1,789 bp (Supplementary Figures S1A,B). BlastX analysis of the insert predicted it to contain five putative ORFs (**Figure 1A**). The hypothetical products of the five ORFs had a homolog with >90% amino acid identity from *Streptococcus* sp. 263_SSPC and *G. adiacens* ATCC 49175. Of the ORFs identified, three encoded a UDP-galactose mutase, sulfurtransferase and amidohydrolase. The remaining ORFs were predicted to encode two half ABC transporters that were named TetA(60) and TetB(60) as their putative amino acid sequences had less than 80% similarity to any other tetracycline resistance protein amino acid sequence and therefore fulfilled the criteria for a new tetracycline resistance gene. Interestingly, in the *Streptococcus* sp. 263_SSPC genome sequence two transposase genes were found 634 bp and 6,196 bp upstream of the UDP-galactose mutase. No such genes were identified in the *G. adiacens* ATCC 49175 genome. Clustal Omega alignments of the putative amino acid sequences of TetA(60) and TetB(60) to characterized antibiotic resistance heterodimeric ABC transporters showed that they were more closely related to TetA(46) and TetB(46) (39.27% and 42.28% identity, respectively) and YheH and YheI (40.93% and 46.61%, respectively). TetAB(60) were less related to the MDR ABC transporters EfrAB, PatAB and LmrCD of *E. faecalis, S. pneumoniae* and *L. lactis*, respectively (≤34.46%), **Table 3**.

**Table 3:** Alignment of TetAB(60) to other antibiotic resistance ABC transporters.

		Percentage similarity	
Source	ABC half transporter	TetA(60)	TetB(60)
Human saliva metagenomic library (this study)	TetA(60)	100%	24.69%
	TetB(60)	24.69%	100%
*Bacillus subtilis* (Torres et al., 2009)	YheH	40.93%	27.11%
	YheI	24%	46.61%
*Streptococcus australis* (Warburton et al., 2013)	TetA(46)	39.27%	24.16%
	TetB(46)	24.69%	42.28%
*Enterococcus faecalis* (Lee et al., 2003)	EfrA	28.62%	27.37%
	EfrB	28.96%	34.46%
*S. pneumoniae* (Baylay et al., 2015)	PatA	25.9%	26.69%
	PatB	23.77%	29.68%
*Lactococcus lactis* (Lubelski et al., 2004)	LmrC	27.69%	26.5%
	LmrD	25.14%	30.07%

Each putative ABC half transporter peptide was predicted to be 579 amino acids and both contained a predicted NBD and a TMD, which are hallmarks of ABC transporters. Additionally, there is a 4 bp overlap of the genes, with the start codon of *tet*B(60) being contained within *tet*A(60), although the genes are in different reading frames.

To determine if these two genes were responsible for the observed tetracycline resistance phenotype of PS9, *tet*A(60) and *tet*B(60) were individually and jointly subcloned into *E. coli* EPI300 using the pHSG396 cloning vector. Only *E. coli*::pHSG396*tet*AB(60) grew on 5 μg/ml of tetracycline, showing that both *tet*A(60) and *tet*B(60) were required for the tetracycline resistance.

In order to ascertain whether the gene products function as a heterodimeric ABC transporter that confers resistance to tetracycline, a 69 and 57 base pair deletion was made to remove the Walker A motif of the NBD from either *tet*A(60) or *tet*B(60), respectively (**Figure 1B**). Both mutants, *E. coli*::pHSG396 *tet*B(60)Δ*tet*A(60) and *E. coli*::pHSG396*tet*A(60)Δ*tet*B(60) were susceptible to tetracycline. This confirmed that the ABC transporter activity of these gene products is responsible for the tetracycline resistance in PS9 and the *E. coli*::pHSG396*tet*AB(60), **Table 4**.

**Table 4:** MICs of tetracycline antibiotics for *E. coli*::pHSG396*tet*AB(60) and mutant strains.

Strain	Tetracycline (μg/ml)	Minocycline (μg/ml)	Tigecycline (μg/ml)
*E. coli*::pHSG396	2	1	0.5
*E. coli*::pHSG396*tet*AB(60)	32	1	8
*E. coli*::pHSG396*tet*B(60)Δ*tet*A(60)	2	1	0.5
*E. coli*::pHSG396*tet*A(60)Δ*tet*B(60)	2	1	0.5

Using the broth dilution method, the MIC of tetracycline for *E. coli* EPI300, *E. coli*::pHSG396, *E. coli*::pHSG396*tet*AB(60), *E. coli*::pHSG396*tet*B(60)Δ*tet*A(60) and *E. coli*::pHSG396 *tet*A(60)Δ*tet*B(60) was determined (**Table 4**). The MIC of tetracycline for *E. coli*::pHSG396*tet*AB(60) was found to be 32 μg/ml. The MICs for the mutants and the controls strains were 16-fold lower at 2 μg/ml. To determine if *tet*AB(60) was able to confer resistance to later generation tetracycline derivatives, MIC assays were conducted using minocycline and tigecycline. The MIC of minocycline for all strains and clones was 1 μg/ml. The MIC of tigecycline for *E. coli*::pHSG396*tet*AB(60) was 16-fold higher than the control and mutant strains at 8 μg/ml, which was above the clinical break point for Enterobacteriaceae (0.5 μg/ml).

Disk diffusion assays were used to discern the spectrum of resistance for this transporter. *E. coli*::pHSG396*tet*AB(60) was less sensitive to tetracycline but equally sensitive to cefotaxime, ceftazidime, metronidazole, neomycin, ciprofloxacin, nalidixic acid, gentamicin, amikacin, amoxicillin/clavulanate and trimetoprim/sulfametoxazole as *E. coli* EPI300 and *E. coli*::pHSG396. *E. coli* EPI300 was intrinsically resistant to erythromycin which has been described previously and attributed to AcrAB-TolC mediated eﬄux and membrane impermeability (Vaara, 1993; Chollet et al., 2004).

**FIGURE 2:**
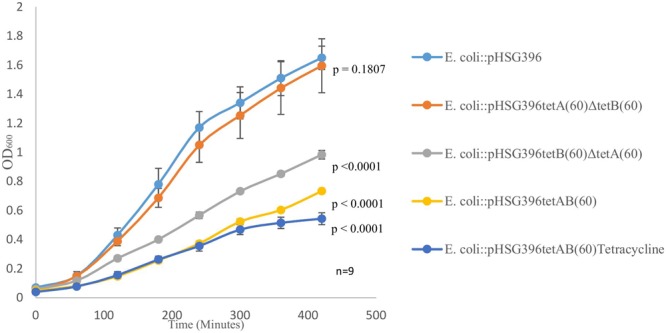
**The above graph depicts growth curves for *E. coli*::pHSG396, *E. coli*::pHSG396*tet*B(60)Δ*tet*A(60) and *E. coli*::pHSG396*tet*A(60)Δ*tet*B(60) grown in LB and chloramphenicol for 7 h.** It also shows the growth curve for *E. coli*::pHSG396*tet*AB(60) grown in LB and chloramphenicol with and without tetracycline to determine how the antibiotic effected the clone’s growth. *P*-values for OD_600_ at 7 h were calculated from biological triplicate OD_600_ measurements at 420 min for each clone compered to *E. coli*::pHSG396 are indicated beside each growth curve.

We observed, when it was first isolated, that PS9 grew slower and formed smaller colonies than *E. coli*::pCC1BAC even in the absence of tetracycline in the growth media; this phenotype was also observed for the *E. coli*::pHSG396*tet*AB(60) subclone. Furthermore, it was noted that the *E. coli*::pHSG396*tet*B(60)Δ*tet*A(60) and *E. coli*::pHSG396*tet*A(60)Δ*tet*B(60) mutants did not have such a large growth defect. Growth curves revealed that although there were significant differences between *E. coli*::pHSG396 and *E. coli*::pHSG396*tet*A(60)Δ*tet*B(60) maximum growth rates (0.993 ± 0.05 and 0.917 ± 0.05, respectively; p = 0.005) there was no significant difference in their OD_600_ of cultures at 7 h (1.65 ± 0.08 and 1.565 ± 0.16; p = 0.1807) when grown in the absence of tetracycline (**Figure 2**).

Compared to *E. coli*::pHSG396, *E. coli*::pHSG396*tet*B(60)Δ*tet*A(60) and *E. coli*::pHSG396*tet*AB(60) reached lower OD_600_ at 7 h when they were grown in the absence of tetracycline (0.983 ± 0.03, 0.733 ± 0.01, respectively, p < 0.0001). Additionally, when grown in the presence of tetracycline, *E. coli*::pHSG396*tet*AB(60) reached an even lower OD_600_ at 7 h (0.543 ± 0.04, p < 0.0001). Whilst the maximum growth rates of *E. coli*::pHSG396*tet*B(60)Δ*tet*A(60) grown without tetracycline and *E. coli*::pHSG396*tet*AB(60) grown without or with tetracycline were not significantly different from each other (0.702 ± 0.06, 0.68 ± 0.03 and 0.692 ± 0.09, respectively; p = 0.435 to 0.879), they were 1.14-1.46 fold lower than the maximum growth rate of *E. coli*::pHSG396 grown without tetracycline (p < 0.0001). We therefore suggest that there is a fitness cost due to the activity of TetAB(60) rather than the carriage of the plasmid itself and that TetB(60) contributes more to this fitness cost than TetA(60).

## Discussion

There has been a resurgence in the use of tetracyclines in human therapy due to the recent development of a number of semisynthetic derivatives of the antibiotic that are efficacious against antibiotic resistant pathogens (Rubinstein and Vaughan, 2005; Anstead et al., 2014; Shen et al., 2015; Lin et al., 2016; Van Berkel et al., 2016). Tigecycline is the first of this new generation of tetracyclines to enter clinical use, being effective against MDR pathogens including carbapenem and colistin resistant microorganisms and those expressing specific tetracycline eﬄux systems and RPPs (Fluit et al., 2005; Cai et al., 2011). However, it is worrisome that resistance to tigecycline has already been described and associated with multidrug eﬄux systems and ribosomal mutations (Villa et al., 2014; Zhong et al., 2014; Lupien et al., 2015).

In this study we characterized a clone, PS9, isolated from a human oral saliva metagenomic library that exhibited high levels of resistance to tetracycline and tigecycline. BlastN alignments of the clone with *Streptococcus* sp. 263_SSPC and *G. adiacens* ATCC 49175 revealed the PS9 insert to have 90% and 92% nucleotide similarity to these species, respectively, indicating a probable Gram-positive origin for the insert. *Streptococcus* spp. are predominant in the oral cavity although to the best of our knowledge *Streptococcus* sp. 263_SSPC has not been identified (Segata et al., 2012). *Granulicatella* spp. including *G. adiacens* are also abundant in the oral cavity, typically inhabiting the mucosa (Aas et al., 2005). Tetracycline resistance mediated by Tn*916* encoded *tet*(M) has been described for oral *Streptococcus* and *Granulicatella* spp. (Lancaster et al., 2005). Additionally, MFS and ABC transporter genes conferring resistance to tetracyclines have also been identified in oral *Streptococcus* spp. including *tet*(L) and *tet*AB(46), respectively (Chen et al., 2013; Warburton et al., 2013). Although tetracycline resistance has been observed in *Granulicatella* spp., minimal characterisation studies have been conducted (Zheng et al., 2004; De Luca et al., 2013). Although *Streptococcus* and *Granulicatella* spp. are abundant in the oral cavity, it is not known how prevalent *tet*AB(60) is in the oral cavity and further work beyond the scope of our characterisation study is required to address this.

Two transposase genes were located up stream of the UDP-galactose mutase gene in *Streptococcus* sp. 263_SSPC, however, it is unknown if these transposases are found in the host genome of the PS9 sequence or if they are capable of transposition of *tet*AB(60). The alignments also identified an inverted region in the PS9 insert when compared to the *Streptococcus* sp. 263_SSPC and *G. adiacens* ATCC 49175 genomes which may have resulted from a DNA breakage followed by repair or a transposition event.

Analysis of the insert revealed it contained five ORFs, predicted to encode a putative UDP-galactose mutase, a sulfurtransferase, an amidohydrolase and two ABC half transporters. Each predicted protein had amino acid sequences with high similarity (>90% identity) to proteins from *Streptococcus* sp. 263_SSPC and *G. adiacens*. Numerous heterodimeric ABC transporters capable of conferring antibiotic resistance have been described, including the MDR transporters EfrAB of *E. faecalis* (Lee et al., 2003), PatAB from *S. pneumoniae* (Baylay et al., 2015), LmrCD from *L. lactis* (Lubelski et al., 2004) and the recently characterized EfrCD of *E. faecalis* (Hurlimann et al., 2016). These transporters have been shown to confer resistance to fluoroquinolones, tetracyclines and biocides among other antimcrobials. Alignment of the putative amino acid sequences of TetAB(60) to other antibiotic resistance heterodimeric ABC transporters showed that they were most closely related to TetAB(46) and YheH/I and less so to the MDR ABC transporters EfrAB, PatAB and LmrCD. As TetAB(46) has been shown to be most closely related to YheH/I this suggested that TetAB(60) was also tetracycline specific (Warburton et al., 2013).

We showed that both *tet*A(60) and *tet*B(60) were required to confer tetracycline resistance in *E. coli* EPI300, suggesting that the product of these genes formed a heterodimeric ABC transporter with each gene product containing a TMD and NBD as revealed by BlastX (Dawson and Locher, 2006). Previous studies have used *E. coli* as a host to characterize Gram-positive antimicrobial ABC transporters including EfrAB from *E. faecalis* and LmrA from *L. lactis* (Lee et al., 2003; Achard-Joris et al., 2005). As *E. coli*::pHSG396*tet*AB(60) did not contain the inverted sequence it is likely that this sequence does not affect expression of *tet*AB(60).

Walker A motifs are found in many ATP utilizing enzymes including ABC transporters and are required for binding and stabilizing ATP (Ramakrishnan et al., 2002). Deletion of these motifs from ATP transporters has been shown to result in a loss of function (Warburton et al., 2013). Individual in-frame deletions of these motifs from either *tet*A(60) or *tet*B(60) led to a loss of the tetracycline and tigecycline resistance phenotype providing further evidence that the products of these genes form a heterodimeric ABC transporter.

Compared to *E. coli*::pHSG396, *E. coli*::pHSG396*tet*AB(60) was 16-fold more resistant to tetracycline (MIC of 32 μg/ml) and tigecycline (MIC of 8 μg/ml). Although *E. coli*::pHSG396*tet*AB(60) showed levels of resistance to tetracycline and tigecycline beyond the EUCAST breakpoints, it was as susceptible to minocycline as *E. coli*::pHSG396, indicating that minocycline is not a substrate for this transporter (Olson et al., 2006; Ramos et al., 2009). Eﬄux mediated tigecycline resistance has been described previously in *Pseudomonas aeruginosa* and *Klebsiella pneumoniae*, being attributed to the activity of an ABC and a resistance nodulation division (RND) transporter, respectively (Dean et al., 2003; He et al., 2015; McDaniel et al., 2016). TetAB(46) was also shown to confer low tigecycline resistance in *S. australis* (Warburton et al., 2013).

TetAB(60) appeared to be specific for tetracycline and tigecycline as disk diffusion assays demonstrated *E. coli*::pHSG396 to be as susceptible as *E. coli*::pHSG396*tet*AB(60) to cefotaxime, ceftazidime, metronidazole, neomycin, ciprofloxacin, nalidixic acid, gentamicin, amikacin, amoxicillin/clavulanate and trimetoprim/sulfametoxazole and erythromycin, providing further evidence for the tetracycline specificity of the ABC transporter.

The observed fitness cost associated with *tet*AB(60) was not observed in either mutant as although *E. coli*::pHSG396*tet*A(60)Δ*tet*B(60) had a lower maximum growth rate than the control it had a comparable final OD_600_ to *E. coli*::pHSG396 and*E. coli*::pHSG396*tet*B(60)Δ*tet*A(60) exhibited faster growth than *E. coli*::pHSG396*tet*AB(60). This indicated that the growth defect was a result of TetAB(60) activity rather than from maintenance of the plasmid. Additionally, as *E. coli*::pHSG396*tet*B(60)Δ*tet*A(60) grew less well than *E. coli*::pHSG396*tet*A(60)Δ*tet*B(60) it suggests that TetB(60) produces a greater cost to the *E. coli* host than TetA(60).

## Conclusion

We have identified two novel genes from the human oral cavity that likely produce a heterodimeric ABC transporter, TetAB(60). TetAB(60) specifically exports tetracycline and tigecycline conferring high levels of resistance to these antibiotics in an *E. coli* host. A limitation of this work is that we do not know the prevalence of these genes in the human oral cavity. Further work should be undertaken to survey its prevalence in various niches, to determine how common these genes are, and their possible clinical relevance for treating bacterial infections with tetracycline derivatives. This work also shows that the human oral cavity harbors unknown tetracycline resistance determinants in the absence of any obvious selection pressure. There is potential for these genes to be acquired by mobile genetic elements and transferred to bacterial pathogens, which is particularly worrying given the recent identification of a carbapenem and colistin resistant strains of *E. coli* some of which could only be inhibited by doxycycline and tigecycline (Liu et al., 2016; Mediavilla et al., 2016; Yao et al., 2016). However, the associated fitness cost of *tet*AB(60) observed in *E. coli* may limit any possible fixation following dissemination of the genes from their native host to *E. coli* strains in the absence of a tetracycline or tigecycline selective pressure.

## Author Contributions

LR contributed to the design of the experiments as well as to the acquisition, analysis, interpretation of the data included in this manuscript, wrote initial and revised drafts of the manuscript and approves of the final manuscript being submitted and also agrees to be accountable for the work detailed in the submitted manuscript. AR conceived the project, contributed to the design of experiments conducted throughout it, interpreted results and contributed to the drafting and revising of the manuscript being submitted and also approved the final draft of the manuscript and agrees to be accountable for the work detailed in the submitted manuscript. MA conceived the project, contributed to the design and direction of experiments within it, interpreted results and made revisions to the final manuscript, approved the manuscript being submitted and also agrees to be accountable for the work represented in the submitted manuscript.

## Conflict of Interest Statement

The authors declare that the research was conducted in the absence of any commercial or financial relationships that could be construed as a potential conflict of interest.
