# Supplementary material for: Eﬄux in the Oral Metagenome: The Discovery of a Novel Tetracycline and Tigecycline ABC Transporter
Source: Front Microbiol. 2016 Dec 6;7:1923. doi: 10.3389/fmicb.2016.01923 (PMC5138185; doi:10.3389/fmicb.2016.01923)
Supplement: Supplementary file 1 [file Data_Sheet_1.docx]

Supplementary Material

**Efflux in the Oral Metagenome:**

**The Discovery of a Novel Tetracycline and Tigecycline ABC Transporter**

**Liam J. Reynolds*^1,2^, Adam P. Roberts^2^, Muna F. Anjum^1,2^**

*** Correspondence:** Liam J. Reynolds: [liam.reynolds.12@ucl.ac.uk](mailto:liam.reynolds.12@ucl.ac.uk)

**Table S1. Primers used in this study**

| **Name** | **Sequence (5’-3’)** | **Information** | **Source** |
| --- | --- | --- | --- |
| **pCC1-F** | GGATGTGCTGCAAGGCGATTAAGTTGG | End sequencing of pCC1BAC | Epicentre® |
| **pCC1-R** | CTCGTATGTTGTGTGGAATTGTGAGC | End Sequencing of pCC1BAC | Epicentre® |
| **M13-F** | GTTTTCCCAGTCACGAC | End sequencing for pHSG396 | Beckman Coulter Genomics |
| **M13-R** | CAGGAAACAGCTATGAC | End sequencing for pHSG396 | Beckman Coulter Genomics |
| **TetRseq1R** | CTTTTCTACGGTGGGTATC | Sequencing of ABC transporter region of PS9 | This Study |
| **TetRseq2R** | GATGCGAAACGGAAAGGG | Sequencing of ABC transporter region of PS9 | This Study |
| **TetRseq3R** | GGCAGAGAACCGTTGGTACG | Sequencing of ABC transporter region of PS9 | This Study |
| **TetRseq4R** | CTCAACAATGAATCGATTC | Sequencing of ABC transporter region of PS9 | This Study |
| **ABC1FH** | GGTGGTAAGCTTGGTGAATGAAGTATAGC | Amplification of ABC transporter genes Introduced HindIII site underlined | This Study |
| **ABC1FB** | GGTGGTGGATCCGGTGAATGAAGTATAGC | Amplification of ABC transporter genes Introduced BamHI site underlined | This Study |
| **ABC1RB** | GGTGGTGGATCCCCTGCTTTGAAATCATGCG | Amplification of ABC transporter genes Introduced BamHI site underlined | This Study |
| **ABC2FH** | GGTGGTAAGCTTGCAATTCGTCATGCGGATG | Amplification of ABC transporter genes Introduced HindIII site underlined | This Study |
| **ABC2RB** | GGTGGTGGATCCCCTCAATCAGTTTCCTAC | Amplification of ABC transporter genes Introduced BamHI site underlined | This Study |
| **ABC2FB** | GGTGGTGGATCCGGTGAATGAAGTATAGC | Amplification of ABC transporter genes Introduced BamHI site underlined | This Study |
| **ABC1delF** | CCGTACCGAGGAGAGAAGCTG | Deletion of Walker A motif from *tet*A(60) | This Study |
| **ABC1delR** | CTCGCCTTTACGTAACG | Deletion of Walker A motif from *tet*A(60) | This Study |
| **ABC2delF** | CGCTTCTACGACCCAACG | Deletion of Walker A motif from *tet*B(60) | This Study |
| **ABC2delR** | CGTCTGTCCTGGTTCTAC | Deletion of Walker A motif from *tet*B(60) | This Study |
