# Supplementary material for: Eﬄux in the Oral Metagenome: The Discovery of a Novel Tetracycline and Tigecycline ABC Transporter
Source: Front Microbiol. 2016 Dec 6;7:1923. doi: 10.3389/fmicb.2016.01923 (PMC5138185; doi:10.3389/fmicb.2016.01923)
Supplement: Supplementary file 2 [file Data_Sheet_2.docx]

Supplementary Material

**Efflux in the Oral Metagenome:**

**The Discovery of a Novel Tetracycline and Tigecycline ABC Transporter**

**Liam J. Reynolds*^1,2^, Adam P. Roberts^2^, Muna F. Anjum^1,2^**

*** Correspondence:** Liam J. Reynolds: [liam.reynolds.12@ucl.ac.uk](mailto:liam.reynolds.12@ucl.ac.uk)

Figure S1. PS9 Sequence Alignments

(a)

UDP Galactopyranose

PS9 319 GTCCATGGTATAATGAATAAGATAAAATAAGAAATGGGGAAACCACATGAAACCATTTGA 378

|||||||||||||| || ||||| ||| ||||||||||||||| ||||||||||||||||

G.adi 88889 GTCCATGGTATAATAAACAAGATTAAACAAGAAATGGGGAAACGACATGAAACCATTTGA 88830

PS9 379 TTACATCGTTGTGGGTGCTGGATTATTCGGTGCGACCTTTGCGCATGAAGCAGCGACACG 438

||| ||||||||||| |||||| ||||||| || || |||||||||||||||||||||||

G.adi 88829 TTATATCGTTGTGGGGGCTGGACTATTCGGAGCAACTTTTGCGCATGAAGCAGCGACACG 88770

PS9 439 CGGTTATAAAGTAAAAGTGATTGAGAAGAGAAATCATATTGCGGGGAATATTTATACAAA 498

||||||||||||||||||||||||||| ||||| ||||||||||||||||||||||||||

G.adi 88769 CGGTTATAAAGTAAAAGTGATTGAGAAAAGAAACCATATTGCGGGGAATATTTATACAAA 88710

PS9 499 AGAAGTGGAAGGCATTCAAGTGCACGAATACGGTGCGCATATTTTCCACACGAGCGACAA 558

|||||||||||||||||||||||| |||||||||||||||||||||||||||||||||||

G.adi 88709 AGAAGTGGAAGGCATTCAAGTGCATGAATACGGTGCGCATATTTTCCACACGAGCGACAA 88650

PS9 559 GAAGATTTGGGATTACGTGCATCAGTTTGCGACATTTAATCGCTATACCAATACTCCAGT 618

|||||||||||||||||||||||||||||||||||||||||||||||| ||||| || ||

G.adi 88649 GAAGATTTGGGATTACGTGCATCAGTTTGCGACATTTAATCGCTATACGAATACACCCGT 88590

PS9 619 TGCCAACTTCAACGGAGAAATCTACAACTTGCCCTTTAACATGAATACTTTCAATAAATT 678

||||||||||||||||||||||||||||||||||||||| |||||||| || ||||||||

G.adi 88589 TGCCAACTTCAACGGAGAAATCTACAACTTGCCCTTTAATATGAATACCTTTAATAAATT 88530

PS9 679 ATGGGGTGTGGTGACGCCACAAGAAGCCGAAGCGAAAATTGCTGAACAGCGTGCGGTACT 738

||||||||| ||||||||||||||||||||||||||||| ||||| |||||||| || |

G.adi 88529 ATGGGGTGTCGTGACGCCACAAGAAGCCGAAGCGAAAATCGCTGAGCAGCGTGCAGTTTT 88470

PS9 739 CGGGGACAAAGAGCCTGAAAACTTAGTAGAGCAGGCGATTTCTCTTGTTGGGGAAGATAT 798

||||||||||||||||||||||||| |||||||||||||||||||||||||||||||||

G.adi 88469 GGGGGACAAAGAGCCTGAAAACTTAGAAGAGCAGGCGATTTCTCTTGTTGGGGAAGATAT 88410

PS9 799 TTACTACAAGCTCATCAAGGGCTATACGGAGAAACAATGGGGACGTTCGGCTACAGAATT 858

||||||||||||||| ||||| || ||||||||||| |||||||||||||||||||||||

G.adi 88409 TTACTACAAGCTCATTAAGGGATACACGGAGAAACAGTGGGGACGTTCGGCTACAGAATT 88350

PS9 859 ACCAGCATTTATCATTCGCCGCTTGCCGGTTCGTTACACTTACGATAACAACTATTTTAA 918

|||||| |||||||| |||||| |||||||||| |||||||||||||||||||| |||||

G.adi 88349 ACCAGCGTTTATCATCCGCCGCCTGCCGGTTCGCTACACTTACGATAACAACTACTTTAA 88290

PS9 919 CGATACGTACCAAGGGATTCCGATTGGTGGCTATACGAAGATGATTGAAGCCATGCTGGA 978

||||||||| ||||||||||| |||||||| |||||||||||||||||||||||||||||

G.adi 88289 CGATACGTATCAAGGGATTCCAATTGGTGGTTATACGAAGATGATTGAAGCCATGCTGGA 88230

PS9 979 CCATGAGAATATCGAAGTCGAGTTGAATGTTGATTTCTTTGCGAAAAAAGATGAGTACTT 1038

||||||||||||||||||||||||||||||||||||||| ||||||||||| ||||||||

G.adi 88229 CCATGAGAATATCGAAGTCGAGTTGAATGTTGATTTCTTCGCGAAAAAAGACGAGTACTT 88170

PS9 1039 AAGTAGTGGTGCCAAAATCGTCTTCACGGGAATGATTGATGAGTTCTTCGATTATGAACT 1098

|| ||||||||| |||||||||||||||||||||||||| |||||||| |||||||||||

G.adi 88169 AAATAGTGGTGCTAAAATCGTCTTCACGGGAATGATTGACGAGTTCTTTGATTATGAACT 88110

PS9 1099 AGGCACATTAGAATACCGTTCGCTCCGTTTTGAAACAGAAGTCGTGGACGTGGAGAACTA 1158

|| || | ||||| ||||| | || |||||||||||||||||||||||||| |||||

G.adi 88109 TGGAACGCTGGAATATCGTTCATTGCGATTTGAAACAGAAGTCGTGGACGTGGAAAACTA 88050

PS9 1159 TCAAGGAAATGCGGTAGTAAACTATACAGACCGCGAAACGCCATACACTCGTATCATCGA 1218

||||||||||||||||||||||||||||||||||||||||||||||||||||| |||||

G.adi 88049 CCAAGGAAATGCGGTAGTAAACTATACAGACCGCGAAACGCCATACACTCGTATTATCGA 87990

PS9 1219 GCATAAGCATTTCGAGTTTGGCACGCAGCCCAAAACGGTGATTACGCGTGAGTATCCTGC 1278

||||||||||||||||||||| || ||||| |||||||||||||||||||||||||||||

G.adi 87989 GCATAAGCATTTCGAGTTTGGGACTCAGCCAAAAACGGTGATTACGCGTGAGTATCCTGC 87930

PS9 1279 CGATTGGAAAGTGGGAGACGAGCCTTATTATCCAGTGAATAATAAAGTCAACAACGACTT 1338

||||||||| ||||| |||||||||||||| |||||||||||| |||| |||||||||||

G.adi 87929 CGATTGGAAGGTGGGCGACGAGCCTTATTACCCAGTGAATAATCAAGTTAACAACGACTT 87870

PS9 1339 GTACGCGCAGTATAAGAAATTAGCGCAGACGGTTCCGCAAGTGATTTTCGGAGGACGCCT 1398

|| ||||||||||| |||||||| ||||||||||| ||||| || ||||| ||||| ||

G.adi 87869 ATATGCGCAGTATAAAAAATTAGCACAGACGGTTCCACAAGTCATCTTCGGTGGACGACT 87810

PS9 1399 TGGACAGTATCGTTATTACGATATGCATCAAGTCATCGCCGCAGCACTAGAAACTGTCGA 1458

|||||||||||||||||||||||||||||||||||||||||| ||||||||||| || ||

G.adi 87809 TGGACAGTATCGTTATTACGATATGCATCAAGTCATCGCCGCTGCACTAGAAACAGT-GA 87751

PS9 1459 TAAA-GAATTCCGTTAAACGCAAGAA-CACCATATTTG 1494

| | ||||| |||| | ||||| |||| ||| ||

G.adi 87750 AACATGAATTTGAGTAAATG-AAGAAACACCCTATATG 87714

Inversion in the Sequence:

PS9 1608 CCGAAGAATCTTGCGGATTTCATAACCACAATAATGGGGAATGGTATTAAATGCGAA-AA 1666

|| ||||||||||||||||||||| ||||||||||||||||| ||| |||| ||| |

G.adi 87499 CCAAAGAATCTTGCGGATTTCATAGCCACAATAATGGGGAATCGTACTAAAGATGAATTA 87558

PS9 1667 AAGCTGGAGTTAGTAATTTATTCCTTACTCCAGCTTTGAGGCTTTAAAGGTGAGAGACTA 1726

||| || | ||| ||||||||||||| || ||||| |||||| |||||||||||||

G.adi 87559 AAG--GGCG-TAGGGATTTATTCCTTACGCCCTTTTTGAAGCTTTAGAGGTGAGAGACTA 87615

PS9 1727 TAGGCTCGAACCGGTACCCCATTATTGTAGTTATGATAAAATCCGCTAGGATTCGTAGGT 1786

|||||||||||||||||||| ||||||||||||||||||||| |||||||||||| |||

G.adi 87616 TAGGCTCGAACCGGTACCCCGTTATTGTAGTTATGATAAAATTCGCTAGGATTCGAAGGA 87675

PS9 1787 ATCA 1790

||||

G.adi 87676 ATCA 87679

Then the sequence continues until the end…

PS9 1822 ATTAAGCATTCACTGTATCTAGTTCTTCGCCGATAGCTT-GAAGACGAGAGACAACCTCT 1880

|||||||||| |||||||||||||||||||||||||||| ||| |||| || || |||

G.adi 87449 ATTAAGCATTTACTGTATCTAGTTCTTCGCCGATAGCTTCTAAG-CGAGCAACGACTTCT 87391

PS9 1881 TCAAGAGATAGGTTGTGTTCTTTCACATAGCGGTTGCGTGGGTGAACACGACATTCATGG 1940

|| || ||||||||||| |||| |||||||||||||||||||||||||| |||||||||

G.adi 87390 TCTAGTGATAGGTTGTGCTCTTGTACATAGCGGTTGCGTGGGTGAACACGGCATTCATGG 87331

PS9 1941 CTGCAACCACGTAAGTATTTGTCTTCGTTCTCTTCTGAAGCTAAGATACGACGGTTACAT 2000

| ||||||||||||||||||||||||||| ||||||||||||||||||||||||||||||

G.adi 87330 CAGCAACCACGTAAGTATTTGTCTTCGTTTTCTTCTGAAGCTAAGATACGACGGTTACAT 87271

PS9 2001 TCTGGGTTTCCACAGTTTACGTAACGTTCGCAAGGTGTACCATCGAACCAGTCTTTCCCA 2060

|||||||| |||||||||||||||||||||||||||| |||||||||||||||||||||

G.adi 87270 TCTGGGTTACCACAGTTTACGTAACGTTCGCAAGGTGAACCATCGAACCAGTCTTTCCCG 87211

PS9 2061 ATTACTACTGGATCTACGTGGTTGATGTCAACAGCGATACGTTCGTCAAATACGTACATT 2120

|| || ||||| ||||||||||||||||||||||||||||| || || ||||| ||||||

G.adi 87210 ATAACCACTGGGTCTACGTGGTTGATGTCAACAGCGATACGCTCATCGAATACATACATT 87151

PS9 2121 TTTCCATCCCATAATTCGCCTTGAACTTCTGGGTCTTTCCCGTAAGTTGCGATACCGCCG 2180

|||||||||||||||||||||||||||||||||||||| ||||||||||||||||| |||

G.adi 87150 TTTCCATCCCATAATTCGCCTTGAACTTCTGGGTCTTTTCCGTAAGTTGCGATACCACCG 87091

PS9 2181 TGTAATTGACCGACATCTTTGTAGCCTTCACGAACCATCCAGCCAGAGAATTTCTCACAG 2240

||||||||||| ||||||||||||||||||||||||||||||||||||||||||||||||

G.adi 87090 TGTAATTGACCAACATCTTTGTAGCCTTCACGAACCATCCAGCCAGAGAATTTCTCACAG 87031

PS9 2241 CGAACGCCACCTGTACAGTAAACAACGACACGTTTGTCCATGAATTTCTCTTTGTTATCG 2300

||||| ||||||||||||||||||||||||||||||||||||||||||||||||||||||

G.adi 87030 CGAACTCCACCTGTACAGTAAACAACGACACGTTTGTCCATGAATTTCTCTTTGTTATCG 86971

PS9 2301 CGAACCCATTGTGGTAATTCACGGAAGTTGCGAATGTCAGGACGGATGGCACCGCGGAAG 2360

||||||||||||||||||||||||||||||||||||||||||||||| ||||||||||||

G.adi 86970 CGAACCCATTGTGGTAATTCACGGAAGTTGCGAATGTCAGGACGGATTGCACCGCGGAAG 86911

PS9 2361 TGACCTAGGTCGTACTCGTAGTCGTTACGAGTGTCTAGTACAACAGTGTTTTCGTCAAGT 2420

|||||||||||||| ||||||||||||||||||||||||||||||||||||||||||||

G.adi 86910 TGACCTAGGTCGTATTCGTAGTCGTTACGAGTGTCTAGTACAACAGTGTTTTCGTCAAGG 86851

PS9 2421 AGGGCTTCTTTAAACTCTTGTGGAGAAAGGTAAGCCCCTGTTGTTTCAAGTGGGTTAATA 2480

|| ||||| || || |||| ||||||||||||||| ||||||||||| | ||||||||||

G.adi 86850 AGTGCTTCCTTGAATTCTTTTGGAGAAAGGTAAGCACCTGTTGTTTCTAATGGGTTAATA 86791

PS9 2481 TCGTTGTCGAAGTCGTTATCTTCTAAACCAAGGTGTACGATTTCTTTTTTGTAACGAACG 2540

||||| ||||||||||| || |||||||||||||||||||||||||||||||||||||||

G.adi 86790 TCGTTATCGAAGTCGTTGTCCTCTAAACCAAGGTGTACGATTTCTTTTTTGTAACGAACG 86731

PS9 2541 AACATCTTCTTGAAGGCTTGCTCGCTTTCTTCGTCGATTTTAAACCATAAGTCTTCCATC 2600

|||||||||||||||||||||||| ||||||||||||||||||||||||||||||||||

G.adi 86730 AACATCTTCTTGAAGGCTTGCTCGTTTTCTTCGTCGATTTTAAACCATAAGTCTTCCATG 86671

PS9 2601 CCTGGAAGAGAGTGAACATAGTCCATATATTTTTGAGTTGTTTCGTAATCACCAGATACC 2660

|| ||||||||||||||||||||||||||||||||||| ||||| ||||||||||| ||

G.adi 86670 CCAGGAAGAGAGTGAACATAGTCCATATATTTTTGAGTAGTTTCATAATCACCAGAAACA 86611

PS9 2661 GTTCCGTTAATTCCTTCGTCAGCGACAAGGATACGTCCTTTTAATCCGATTGATTTGCAG 2720

|| ||||| ||||||||||||||||||||||||||||||||||||||||||||||||||

G.adi 86610 GTCCCGTTGATTCCTTCGTCAGCGACAAGGATACGTCCTTTTAATCCGATTGATTTGCAA 86551

PS9 2721 AATGCTAAATGATCTGCCGCGAATTGTTCCGCATTTTCGATTGGAACATATTTATAGTAA 2780

||||||||||||||||| ||||||||||||||||| ||||| ||||| |||||||| |||

G.adi 86550 AATGCTAAATGATCTGCAGCGAATTGTTCCGCATTCTCGATGGGAACGTATTTATAATAA 86491

Sulfurtransferase

PS9 2781 AGTAAAACACGAATGTCTTTTGCCATATTGTAAATCTTTCATCCTTTTCTAATTTGTAAT 2840

|||||||||||||| ||||||||||||||||| ||||||||||||||||| ||||||||

G.adi 86490 AGTAAAACACGAATATCTTTTGCCATATTGTAGTTCTTTCATCCTTTTCTAGTTTGTAAT 86431

PS9 2841 GATTCTATTGTAACTGTATTGAGAATGATTATCAATGAATTCGATTGTGAAAAAGTTCGA 2900

| |||||||||||| |||||||||| |||||||||||||||||||||||||| |||||

G.adi 86430 GGTTCTATTGTAACCGTATTGAGAACCATTATCAATGAATTCGATTGTGAAAATCTTCGA 86371

PS9 2901 AAGGTATTGACATGAC-GGTATTTTACGGTATAATT-CAGATATAACGTTAAATTT-ATC 2957

|||||||||||||||| | | |||| ||||||||| || || |||| | ||||||

G.adi 86370 AAGGTATTGACATGACAGCGA-TTTAAGGTATAATTTCAAATGTAACATAAAATTTTGAG 86312

PS9 2958 AGATTGGAGGAGAAATCAATGAACAACTTAAACGTAAAACAAGT-ACAAGATTTGAGAAA 3016

|||||||||||||||||||||||||||||| | ||||| ||| | ||| ||| ||

G.adi 86311 AGATTGGAGGAGAAATCAATGAACAACTTAGTAGAAAAACGAGTGAATAGA-ATGATGAA 86253

PS9 3017 CCTTCCAATTTTGGTGAATGAAGTATAGCGCATTTTTATAGATAAGTTTA--AGTG-AGC 3073

|||||||||| |||| | | |||||||||||||| ||||| || ||| | || |||

G.adi 86252 CCTTCCAATTCAAGTGATTCAGTTATAGCGCATTTTTTTAGATGAGATTAGGAATGAAGC 86193

PS9 3074 CGTTACTTTTTCCATGCGTGGGGAAGGTGACGGCTTTTTT-GATGGAGGAAAACGATGAA 3132

*tet*A(60)

||||||||||||||||||||||||||||||||||||||| |||||||||||||||||||

G.adi 86192 TGTTACTTTTTCCATGCGTGGGGAAGGTGACGGCTTTTTTTGATGGAGGAAAACGATGAA 86133

PS9 3133 CGATTTATTAAAAGTCATTATTAATTTTATAAAGAAACATCCGATGCGCTACCTTGTTAG 3192

||||||| ||||| | | || |||||||| ||||||||||||||||| ||||||||

G.adi 86132 CGATTTAATAAAATTAGTGATAGGCTTTATAAAAAAACATCCGATGCGCTATCTTGTTAG 86073

PS9 3193 TTTTATTTTGATGATCGGAAGTAGTATTGCGGCGGTGTACCCAGCGCGTATTATCGGACA 3252

|||| | |||||| | | |||||||||||| ||||| || || || | ||| | |||||

G.adi 86072 TTTTGTATTGATGGTTGCAAGTAGTATTGCTGCGGTTTATCCCGCTAGAATTGTGGGACA 86013

PS9 3253 AGTTGTTGATAAAATCGTAGCGAGCGAACTGAATGCCGAGTGGCTTGGGACACAACTCGT 3312

||| || ||||||||||| ||||| |||||||| | ||| ||||| |||| ||||||||

G.adi 86012 AGTAGTCGATAAAATCGTCGCGAGTGAACTGAACGGCGATTGGCTCTGGACGCAACTCGT 85953

PS9 3313 GATTTTAGTCGGGATTATTCTTGTGGCGTATATTACGGAGAGTATTTGGACATATTTTAT 3372

||||||||| ||||||||||| ||||| |||||||||||||||||||||||| |||| ||

G.adi 85952 GATTTTAGTAGGGATTATTCTCGTGGCCTATATTACGGAGAGTATTTGGACAAATTTGAT 85893

PS9 3373 TTTTATTGGGTATTATGAAATTCAAAAAGAATTACGTGTGAAGTTACTACGTAATAATTT 3432

|||||| || ||||||||||| ||||| ||||| |||||||| ||| | | ||||||||

G.adi 85892 TTTTATGGGCTATTATGAAATGCAAAAGGAATTGCGTGTGAAATTATTGCAGAATAATTT 85833

PS9 3433 ACGGAAGAAAATTCCGTTTTATGCGCATTTTAGAACGGGCGAAATTATTACGCGTAGCAG 3492

||||||||||||||||||||||||||||||||| || || |||||||| ||||||||

G.adi 85832 GATGAAGAAAATTCCGTTTTATGCGCATTTTAGAACAGGGGATATTATTACCCGTAGCAG 85773

PS9 3493 TGAAGACGTTACAACGATTGGCGATATGATGGGGTTTGGGATGTTTGCATTGATGAACTC 3552

|||||| || | ||||||||||||||||||||||| || ||||||||| | ||||||||

G.adi 85772 TGAAGATGTGATGACGATTGGCGATATGATGGGGTTCGGAATGTTTGCACTCATGAACTC 85713

PS9 3553 TACATTGCTGATGAGCGTATCGATTTATATGATGGTCACAACGATTTCATTGCCACTGAC 3612

|||||| || ||| |||||||||||||||||||||||||||||||| |||||||||||

G.adi 85712 AACATTGATGGTGACGGTATCGATTTATATGATGGTCACAACGATTTCGTTGCCACTGAC 85653

PS9 3613 CATCGCAGCGATTTTGCCACTGCCAATCCTTTCGTATCTTGTATATAAATGGGGATTCGA 3672

|||| ||| |||| ||||| ||||||||||| ||||| || |||||||||||||| ||

G.adi 85652 TATCGTTGCGGTTTTACCACTTCCAATCCTTTCATATCTCGTGTATAAATGGGGATTTGA 85593

PS9 3673 TTTAGAAGAAGAGTACAACAAGGCGCAAAATGCAGTTTCACAATTAAATAATGAAGTGCT 3732

| | |||||||||||||||||||| |||||||||||||| ||||||||||||||||| ||

G.adi 85592 TCTCGAAGAAGAGTACAACAAGGCACAAAATGCAGTTTCGCAATTAAATAATGAAGTACT 85533

PS9 3733 TGAGATGATTGACGGGACGTATGTGATTCGTGCTTACGGGCAAGAAGATGCGATGATGGA 3792

|||||||||||||| || || || ||||| |||||||||||||||||||||||||||||

G.adi 85532 GGAGATGATTGACGGTACCTACGTCATTCGAGCTTACGGGCAAGAAGATGCGATGATGGA 85473

PS9 3793 TGAGTTCAGGGCGAAAACGAAAAAGGCCATGAAACAAAATATTATCGTGACTGAAATTGA 3852

|||||| | ||||||||||||||||| |||||||||||||| || ||| | ||||||||

G.adi 85472 TGAGTTTCGTGCGAAAACGAAAAAGGCTATGAAACAAAATATCATTGTGTCAGAAATTGA 85413

PS9 3853 ATCGCGCTTTATTCCACTGGCGCAATTATTTATGATGATTAGCTTTACCATTGCCCTTTT 3912

||| |||||||||||| |||| ||||||||||||||||| |||||||||||||| ||| |

G.adi 85412 ATCTCGCTTTATTCCATTGGCACAATTATTTATGATGATCAGCTTTACCATTGCACTTCT 85353

PS9 3913 CTACGGTGGGTATCTAGTATCGACTGGGGCTATTCTAGTCGGGGATGTCATTGCCTTCCA 3972

||||||||| || |||||||||||||| ||||||| |||||||||||||||||||| ||

G.adi 85352 CTACGGTGGATACTTAGTATCGACTGGGACTATTCTCGTCGGGGATGTCATTGCCTTTCA 85293

PS9 3973 AGTCTATATGGGGGCGATTATGTGGCCGATGTTTATGATTGGCGATATTATTACGAACTA 4032

||| ||||||||||| ||||||||||||||||||||||||||||||||||| ||||||||

G.adi 85292 AGTGTATATGGGGGCTATTATGTGGCCGATGTTTATGATTGGCGATATTATCACGAACTA 85233

PS9 4033 TAAACGCGGAAAAGTGGCGACGGAGCGTATTAATGAAGTGTTGAAACATGACGATGAAAT 4092

||||||||||||||||| || ||||||||||||||| | || | | |||||||||| ||

G.adi 85232 CAAACGCGGAAAAGTGGCAACCGAGCGTATTAATGAAATATTAAGATATGACGATGACAT 85173

PS9 4093 TGAACGCGGCGGTACAAAAACGCTCGAGACGATTGAATCCATTGAGTTTAAGGACTTCCA 4152

||||| || || |||||| ||||||||||||||||||| || |||||||| ||||||||

G.adi 85172 CGAACGTGGTGGAACAAAAGCGCTCGAGACGATTGAATCGATAGAGTTTAAAGACTTCCA 85113

PS9 4153 TTTTATGTATCCAGGCGAAGAGGCACCATTATTAAAAGAGATTAACCTTACGTTACGTAA 4212

||||| ||||||||| |||||||| | |||||||||| ||||| ||||| || ||

G.adi 85112 TTTTACGTATCCAGGAGAAGAGGCGTCCTTATTAAAAGGAATTAATCTTACTTTGAAAAA 85053

PS9 4213 AGGCGAGACGCTTGGAATCGTTGGAAAAACGGGTTCTGGGAAGACGACGCTCTTGATGCA 4272

||||||||| ||||||||||| || ||||| |||||||| || || ||||||||||||||

G.adi 85052 AGGCGAGACTCTTGGAATCGTGGGGAAAACAGGTTCTGGAAAAACAACGCTCTTGATGCA 84993

PS9 4273 ATTATTACATCAATTTCCGTACCGAGGAGAGAAGCTGCTCATTAACGGAGAGCCATTGAT 4332

||||||||| ||||| || ||| |||||| ||||| | ||||||| ||| || |||||

G.adi 84992 ATTATTACACCAATTCCCCTACAAAGGAGAAAAGCTCTTTATTAACGCAGAACCGTTGAT 84933

PS9 4333 TGATTACGACACTCAATCGGTGGCAGGGCATCTAGCCTATGTGCCACAAGAACACACCCT 4392

||||||||| | |||| |||||||||||||| | |||||||| |||||||| || || ||

G.adi 84932 TGATTACGATAGTCAAACGGTGGCAGGGCATTTGGCCTATGTACCACAAGAGCATACACT 84873

PS9 4393 TTTCTCACGCACGATTCGCGAGAATATGTTATTCGGAAAAGAGGATGCAACGGATGATGA 4452

|||||||||||||||||| |||||||||||||||||||||||||||||||||||||||||

G.adi 84872 TTTCTCACGCACGATTCGTGAGAATATGTTATTCGGAAAAGAGGATGCAACGGATGATGA 84813

PS9 4453 AATTTGGGAAGCGTTGACGCTAGCCTCTTTTGAAGGAGACGTGAAACGAATGCCAGACGA 4512

||||||||||||||||||||||||||||||||||||||||||||||||||||||||||||

G.adi 84812 AATTTGGGAAGCGTTGACGCTAGCCTCTTTTGAAGGAGACGTGAAACGAATGCCAGACGA 84753

PS9 4513 GCTCGATACGATGGTCGGAGAAAAAGGGGTATCGCTCAGTGGAGGTCAAAAACAACGCTT 4572

||||||||||||||||||||||||||||||||||||||||||||| ||||||||||| ||

G.adi 84752 GCTCGATACGATGGTCGGAGAAAAAGGGGTATCGCTCAGTGGAGGGCAAAAACAACGATT 84693

PS9 4573 ATCGATTGCTCGTGCTTTCTTACGCAACCGTGAATGCTTAATTTTGGATGATGCGTTATC 4632

||||||||| |||||||||||||||||||||||||| ||||| |||||||||||||||||

G.adi 84692 ATCGATTGCGCGTGCTTTCTTACGCAACCGTGAATGTTTAATATTGGATGATGCGTTATC 84633

*L. buchneri* CD034 regions underlined

PS9 4633 TGCAGTTGATGCGAAAACGGAAAGGGAAATTATCTCGCACTTGCAACAAGAACGCGGAGG 4692

||||||||||||||||||||||||||||||||||||||||||||||||||||||||||||

G.adi 84632 TGCAGTTGATGCGAAAACGGAAAGGGAAATTATCTCGCACTTGCAACAAGAACGCGGAGG 84573

PS9 4693 TTGTATGAATATCATTTCTGCGCACAGACTTTCTGCAATTCGTCATGCGGATGAAATTAT 4752

||||||||||||||||||||||||||||||||||||||||||||||||||||||||||||

G.adi 84572 TTGTATGAATATCATTTCTGCGCACAGACTTTCTGCAATTCGTCATGCGGATGAAATTAT 84513

PS9 4753 TGTGATGAATGAAGGACGTATTAGTGAGAGGGGTACCCACGAGGAGCTGCTCGAACAACG 4812

||||||||||||||||||||||||||||||||||||||||||||||||||||||||||||

G.adi 84512 TGTGATGAATGAAGGACGTATTAGTGAGAGGGGTACCCACGAGGAGCTGCTCGAACAACG 84453

PS9 4813 AGGATGGTACTATGAACAGTATCTCACACAAGAAATGGAGGAGGAAATCGAATGAGAACG 4872

*tet*B(60)

||||||||||||||||||||| ||||||||||||||||||||||||||||||||||||||

G.adi 84452 AGGATGGTACTATGAACAGTACCTCACACAAGAAATGGAGGAGGAAATCGAATGAGAACG 84393

PS9 4873 ATGAAGCGATTATTAAGCTACCTCCGTTATGAGAAAAAAGGAGTTCTGATTGGACTCTTC 4932

||||||||||||||||||||||||||||||||||||||||||||||||||||||||||||

G.adi 84392 ATGAAGCGATTATTAAGCTACCTCCGTTATGAGAAAAAAGGAGTTCTGATTGGACTCTTC 84333

PS9 4933 TGCCTGTTACTCTCAACAGGAGCAACCTTAACAGGGCCACTCGTTGCGAAACACATTATC 4992

||||||||||||||||||||||||||||||||||||||||||||||||||||||||||||

G.adi 84332 TGCCTGTTACTCTCAACAGGAGCAACCTTAACAGGGCCACTCGTTGCGAAACACATTATC 84273

PS9 4993 GATAATGTGATTACTCCGATGGGGCAGGCGCATGATTTCAAAGCAGGTGGCCTACTTTTA 5052

|||||||||||||||||||||||||||||||||||||||||||||||||| |||||||||

G.adi 84272 GATAATGTGATTACTCCGATGGGGCAGGCGCATGATTTCAAAGCAGGTGGTCTACTTTTA 84213

PS9 5053 TGGGTTGGTATTTACGTGACAGTGAACTTAGTGGGAGTTGCTGGGGCCTATTTAAACCGT 5112

||||||||||| ||||||||||||||||||||||||||||||||||||||||||||||||

G.adi 84212 TGGGTTGGTATATACGTGACAGTGAACTTAGTGGGAGTTGCTGGGGCCTATTTAAACCGT 84153

PS9 5113 GTATATATGAAGACTCTCTCAAACCGTATTGCAAAACGCATTCGTGACGAAGTGTTCGAG 5172

|||||||||| ||||||||||||||||||||||||||| ||||| |||||||||||||||

G.adi 84152 GTATATATGAGGACTCTCTCAAACCGTATTGCAAAACGTATTCGCGACGAAGTGTTCGAG 84093

PS9 5173 CATGTACAAACCTTGCCGGTATCGTACTTCGACCATTTACCAGCTGGGAAAGTGGTTTCT 5232

||||||||||||||||||||||||||||||||||||||||||||||||||||||||||||

G.adi 84092 CATGTACAAACCTTGCCGGTATCGTACTTCGACCATTTACCAGCTGGGAAAGTGGTTTCT 84033

PS9 5233 AGAATTACAAGTGATACAGAATCAGTGCGTGCAAACTTCTACGTGAGTGGGATTTCGACG 5292

||||||||||||||||||||||||||||||||||||||||||||||||||||||||||||

G.adi 84032 AGAATTACAAGTGATACAGAATCAGTGCGTGCAAACTTCTACGTGAGTGGGATTTCGACG 83973

PS9 5293 CTTTTCAGTACGATTGTGATGTTGGTAGGTGTTTATATAACCATTTTCTTATTAAACGCA 5352

||||||||||||||||||||||||||||||||||||||||||||||||||||||||||||

G.adi 83972 CTTTTCAGTACGATTGTGATGTTGGTAGGTGTTTATATAACCATTTTCTTATTAAACGCA 83913

PS9 5353 ACGCTAGGACTCGTATTATTATTCCTAGTTCCTGTGATGATTCTATGGCAGAGAACCGTT 5412

||||||||||||||||||||||||||||||||||||||||||||||||||||||||||||

G.adi 83912 ACGCTAGGACTCGTATTATTATTCCTAGTTCCTGTGATGATTCTATGGCAGAGAACCGTT 83853

PS9 5413 GCTACGAAGCAGAAAAAATACTATTCCGAAAATCGTGAACTCTATAGTCAGTTGAGTGGA 5472

|||||||||||||||||||||||||||||||||||||||||||| |||||||||||||||

G.adi 83852 GCTACGAAGCAGAAAAAATACTATTCCGAAAATCGTGAACTCTACAGTCAGTTGAGTGGA 83793

PS9 5473 CAATTAAACGAAAGCATTCAAGGAGCAGGCATCGTTCAAGCCTTCCAGCAAGAAGAAAAA 5532

|||||||||||||||||||||||||||||||||||||||||||||||| |||||||||||

G.adi 83792 CAATTAAACGAAAGCATTCAAGGAGCAGGCATCGTTCAAGCCTTCCAGAAAGAAGAAAAA 83733

PS9 5533 ATTGTTGCAGAATATGATGCCACGGCAACTTCTTGGGTAGAAGTTGGTCGTAAGGAATTA 5592

|||||| |||||||||| || || |||||||||||| ||||| |||||||||||||||||

G.adi 83732 ATTGTTTCAGAATATGAAGCAACTGCAACTTCTTGGCTAGAAATTGGTCGTAAGGAATTA 83673

PS9 5593 ATTCTTGAGTCGTACTTCTCGTGGAGTCTTGTCGGCATGCTTCGAAACATTACTCATTTT 5652

||||||||||||||||||||||||||||||||||||||||||||||||||||||||||||

G.adi 83672 ATTCTTGAGTCGTACTTCTCGTGGAGTCTTGTCGGCATGCTTCGAAACATTACTCATTTT 83613

PS9 5653 GGAGTTATCTATTATTTCAGTATGCAGTTTATCGGTGGAACACTCGGGATTTCAGCAGGT 5712

||||||||||||||||||||||||||||||||||||||||||||||||||||||||||||

G.adi 83612 GGAGTTATCTATTATTTCAGTATGCAGTTTATCGGTGGAACACTCGGGATTTCAGCAGGT 83553

PS9 5713 CTTTTATATGCATTTATTGACTACATTAATCGTATATATGAGCCGATTCAAACTTTTATG 5772

||||||||||| ||||||||||||||||| ||||||||||| ||||||||||||||||||

G.adi 83552 CTTTTATATGCGTTTATTGACTACATTAACCGTATATATGAACCGATTCAAACTTTTATG 83493

PS9 5773 AATGTCGTGTCTGGCTTCCAGCAATCAATGGCTGCTGGTGACCGTGTGTTTGAACTAATG 5832

|||||||| || || ||||| |||||||||||||| ||||| ||||| || ||| |||||

G.adi 83492 AATGTCGTATCAGGTTTCCAACAATCAATGGCTGCCGGTGATCGTGTATTCGAATTAATG 83433

PS9 5833 GATACACCGAGCGAGGAATCGGGAGAAGAGCTCTTCACGTTTGATGAAGGATGTATCGAA 5892

|||||||| |||||||| || |||||||||||||||||||||||||||||| | || |||

G.adi 83432 GATACACCAAGCGAGGATTCAGGAGAAGAGCTCTTCACGTTTGATGAAGGACGGATTGAA 83373

PS9 5893 TTTAAAGATGTGAGTTTTGAGTACACAGCGGGAGTTCCTGTGTTGAAACACTTGAATTTC 5952

|||||||||||||| |||| ||| ||| | | |||||||||||||||||||||||||||

G.adi 83372 TTTAAAGATGTGAGCTTTGCGTATACACCTGACGTTCCTGTGTTGAAACACTTGAATTTC 83313

PS9 5953 ACGGTAGAACCAGGACAGACGGTTGCTTTTGTCGGACATACTGGTTCAGGGAAATCATCC 6012

||||||||||| || |||||||| || |||||||| ||||||||||||||||||||||||

G.adi 83312 ACGGTAGAACCTGGGCAGACGGTGGCCTTTGTCGGTCATACTGGTTCAGGGAAATCATCC 83253

PS9 6013 ATTATGAACTTACTGTTTCGCTTCTACGACCCAACGAGTGGTGCTATCTTCATCGATGGC 6072

||||||||||||||||||||||||||||| |||||||||||||||||||||||||||||

G.adi 83252 ATTATGAACTTACTGTTTCGCTTCTACGATCCAACGAGTGGTGCTATCTTCATCGATGGA 83193

PS9 6073 AAAAACACACGCGACTTTAACAGACGTAGTGTTCGAAGCGAGATGGGAATCGTTCTTCAA 6132

||||||||||||||||||||||||| ||||||||||||||||||||||||||||||||||

G.adi 83192 AAAAACACACGCGACTTTAACAGACATAGTGTTCGAAGCGAGATGGGAATCGTTCTTCAA 83133

PS9 6133 GATCCGTACCTCTTTACAGGAACGATTGCTTCAAACGTAGGGCTCAACAATGAATCGATT 6192

||||||||||||||||||||||||||||||||||||||||||||||||||||||||||||

G.adi 83132 GATCCGTACCTCTTTACAGGAACGATTGCTTCAAACGTAGGGCTCAACAATGAATCGATT 83073

PS9 6193 GAGCCTGAGACGATAAAAGAGGCGATTATTAAAGTGGGTGGAGGACATCTACTTACGAAG 6252

||||||||||||| ||||||||| | |||||||| || ||||| |||||||| ||||||

G.adi 83072 CAGCCTGAGACGATTAAAGAGGCGCTCATTAAAGTAGGCGGAGGCCATCTACTCACGAAG 83013

PS9 6253 AGTGACAAGGGCTTGGACTACGAGGTGAAAGAAAAAGGAATGGATTTCTCTTCAGGAGAA 6312

||||||||||| | |||||||||||||| ||||||||||||||||| ||||| ||||||

G.adi 83012 AGTGACAAGGGACTCGACTACGAGGTGAAGGAAAAAGGAATGGATTTTTCTTCCGGAGAA 82953

PS9 6313 CGCCAACTGATTTCATTTGCTCGTGCGATCGTCTTTGACCCGAAAATCTTAATTTTAGAC 6372

|| ||| ||||||||||||||||||||||||||||||||||||||||||||||||||||

G.adi 82952 CGTCAATTGATTTCATTTGCTCGTGCGATCGTCTTTGACCCGAAAATCTTAATTTTAGAT 82893

PS9 6373 GAGGCAACTTCGCATATCGATACCGAGACAGAAGAGATTATTCAGAATGCAATTAATGTC 6432

|| || ||||||||||||||||||||||| ||||| ||||||||||||||||||||||||

G.adi 82892 GAAGCGACTTCGCATATCGATACCGAGACGGAAGAAATTATTCAGAATGCAATTAATGTC 82833

PS9 6433 GTCAAAGAAGGGCGTACAACCTTTATGATTGCTCACCGACTTTCCACAATTGCTCATGCT 6492

|| ||||||||||||||||||||||||||||||||||||||||| || ||||||||||||

G.adi 82832 GTAAAAGAAGGGCGTACAACCTTTATGATTGCTCACCGACTTTCTACCATTGCTCATGCT 82773

PS9 6493 GACCAAATTTTTGTGTTGGATAAAGGAGAAATTGTAGAACGTGGAACGCATGATGAATTG 6552

||||||||||||||||||||||||||||||||||| |||||||||||||||||||||||

G.adi 82772 GACCAAATTTTTGTGTTGGATAAAGGAGAAATTGTGGAACGTGGAACGCATGATGAATTA 82713

PS9 6553 CTTCAACTGCAAGGTCAATATGCCGAAATGGTCGCCCTACAAAAAGGATAATTT-TAAAA 6611

||||||||||||||||||||||||||||||||||| || |||||||| |||| | |||||

G.adi 82712 CTTCAACTGCAAGGTCAATATGCCGAAATGGTCGCACTTCAAAAAGGCTAATGTATAAAA 82653

PS9 6612 AATACAAGACGCTGTTTCCTTTGAAACAGTGTCTTTTCTTTCGTAGCGGTAAACTTTATT 6671

|||| ||||||||||||||||||||||||||||||||||||||| || |||||||||

G.adi 82652 AATATAAGACGCTGTTTCCTTTGAAACAGTGTCTTTTCTTTCGTTGCAAACAACTTTATT 82593

PS9 6672 TTTTTAGTGTTTTTTCAAGGGAATTCATGGTGCATTACGCCTCTTAGTTTGTTACAATGT 6731

||||||||||||||||||||||||||||||||||||||||||||||||||||||||||||

G.adi 82592 TTTTTAGTGTTTTTTCAAGGGAATTCATGGTGCATTACGCCTCTTAGTTTGTTACAATGT 82533

Hydrolase

PS9 6732 GTAGGAAACTGATTGAGGAGAGAACGTATGATTACGAAAGAAGTTATTGAACGTGCAACA 6791

||||||||||||||||||||||||||||||||||||||||||||||||||||||||||||

G.adi 82532 GTAGGAAACTGATTGAGGAGAGAACGTATGATTACGAAAGAAGTTATTGAACGTGCAACA 82473

PS9 6792 ACGCACCGCCGCCATTTACATATGTATCCAGAAGTATCTGGAGAAGAAGTTGAAACAACG 6851

|||||| |||| |||||||||||||||||||||||||| ||||||||||||||||||||

G.adi 82472 ACGCACAGCCGTCATTTACATATGTATCCAGAAGTATCCGGAGAAGAAGTTGAAACAACA 82413

PS9 6852 CGCTATATCCGAGAAGCCCTAGAAGCGATGGGACTTACTTGCTGGAATTTAAAGTCTAAG 6911

|||||||| || ||| || ||||||||||||| ||| || ||||||||| |||| ||

G.adi 82412 CGCTATATACGCGAAACCTTAGAAGCGATGGGGCTTGTCTGTTGGAATTTACAGTCAAAA 82353

PS9 6912 ACGGGAGTGGTTGCAGAACTCGGAAACGGTGAGGGACCAACATTAGCCTTACGTGCCGAC 6971

|| ||||| || |||||| | ||||| |||||||| |||| ||||||||||||||| |||

G.adi 82352 ACAGGAGTCGTCGCAGAAATTGGAAATGGTGAGGGGCCAATATTAGCCTTACGTGCTGAC 82293

PS9 6972 ATCGATGCGCTACCGATTGTGGAACAAACAGGATTAGACTACGCTTCAAAAAACGAAGGA 7031

||||| || |||||||||| ||||||||||||||||| || ||||| ||||| ||||||

G.adi 82292 ATCGACGCTTTACCGATTGTAGAACAAACAGGATTAGATTATGCTTCGAAAAATGAAGGA 82233

PS9 7032 GCGATGCATGCATGCGGACATGATTTCCATACCGCTAGCTTGCTGGGAGCCGTTCAAGTA 7091

||||||||||| || |||||||||||||||||||| ||||||||||||||| ||||||||

G.adi 82232 GCGATGCATGCGTGTGGACATGATTTCCATACCGCAAGCTTGCTGGGAGCCATTCAAGTA 82173

PS9 7092 TTAAAGGCTCAAGAAGACAAATTACAAGGGAAAGTTCGTTTTATTTTCCAACCAGCCGAA 7151

||||||||||||||||||||||||||||||||||||||||||||||||||||||||||||

G.adi 82172 TTAAAGGCTCAAGAAGACAAATTACAAGGGAAAGTTCGTTTTATTTTCCAACCAGCCGAA 82113

PS9 7152 GAAAGTAACCGTGGAGCACGCGCATTGATTTCTGAAGGTGTGCTTGATGGAGTGGATGCG 7211

|||||||||| ||||||||||| |||||||||||||| |||||||| |||||||| |||

G.adi 82112 GAAAGTAACCAAGGAGCACGCGCTTTGATTTCTGAAGGCGTGCTTGAGGGAGTGGACGCG 82053

PS9 7212 ATTATTGGATTCCATAATAAGCCAGAACTTCCAGTTGGAACGATTGGTGTGAAGGAAGGA 7271

||||||||||||||||||||||||||||||||||||||||||||||||||||||||||||

G.adi 82052 ATTATTGGATTCCATAATAAGCCAGAACTTCCAGTTGGAACGATTGGTGTGAAGGAAGGA 81993

PS9 7272 CCTCTGATGGCAGCGGTCGGCCAGTTTAAAGCCGAAATTACAGGGGTAGGAACGCACGCT 7331

|||||||||||||| |||||||||||||||||||||||||||||||||||||||||||||

G.adi 81992 CCTCTGATGGCAGCAGTCGGCCAGTTTAAAGCCGAAATTACAGGGGTAGGAACGCACGCT 81933

PS9 7332 GCAGCACCGCATAACGGAAACGACCCCATCGTTACGGCTTGCCAAGTGATTGCAAATGCT 7391

||||||||||||||||||||||||||||||||||||||||||||||||||||| || ||

G.adi 81932 GCAGCACCGCATAACGGAAACGACCCCATCGTTACGGCTTGCCAAGTGATTGCTAACGCC 81873

PS9 7392 CAAGCGATTGTTGCACGTCATACATCCCCACTTGAACCAGTGGTATTAAGCGTATCGCAT 7451

||||||||||| || |||||||| || |||||||||||||||||||||||||||||||||

G.adi 81872 CAAGCGATTGTCGCTCGTCATACTTCTCCACTTGAACCAGTGGTATTAAGCGTATCGCAT 81813

PS9 7452 ATCGAAGCAGGGAATACATGGAATGTGATTCCAGAGAAAGTATTTTTTGAAGGAACGATT 7511

|||||||||||||||||||||||||||||||| |||||||||||||||||||||||||||

G.adi 81812 ATCGAAGCAGGGAATACATGGAATGTGATTCCGGAGAAAGTATTTTTTGAAGGAACGATT 81753

PS9 7512 CGTACGTTTAATAAAGAAGTGGAACGTCAAATGACGCAGCAATTCGAAAAGATGATTGTT 7571

|| || |||||||||||||| || || ||||||||| |||||||||| ||||||||||||

G.adi 81752 CGCACCTTTAATAAAGAAGTTGAGCGACAAATGACGGAGCAATTCGAGAAGATGATTGTT 81693

PS9 7572 CAAACGGCTGATGTTTACGGGCAAAAAGGAAGTATCGAATGGATTTTAACGCCGCCAGTT 7631

||||| || ||||| || || |||||||||||||| |||||||||||||||||||||||

G.adi 81692 CAAACAGCGGATGTATATGGACAAAAAGGAAGTATTGAATGGATTTTAACGCCGCCAGTC 81633

PS9 7632 GTGCATAATGACGTAGAAATTACAAAAGTAGTGAGACGCACAACGGAGAAATTTGCGACA 7691

|||||||||||||||||||||||||||||||||||||| ||||| ||||||||||| |||

G.adi 81632 GTGCATAATGACGTAGAAATTACAAAAGTAGTGAGACGTACAACTGAGAAATTTGCAACA 81573

PS9 7692 GTGGTGACACCTGAAGTGACTTTAGGAGCAGAAGATTTCGCCAATTATATGGAACACGTT 7751

||||| || || |||| |||||||||||||||||||| ||||||||||||||||| |||

G.adi 81572 GTGGTAACCCCACAAGTAACTTTAGGAGCAGAAGATTTTGCCAATTATATGGAACATGTT 81513

PS9 7752 CCAGGATGTTTTGT 7765

||||||||||||||

G.adi 81512 CCAGGATGTTTTGT 81499

(b)

Strep_263  1     ACTCGTCATTATTGGCGCCTATATGATTGTAAAATCAAGAAAAGCAAAGCGAAATTTATA  60

             |||||||||||||| ||| |||||||||||||||||||||||||||||||||||||||||

PS9 Clone  1     ACTCGTCATTATTGACGCATATATGATTGTAAAATCAAGAAAAGCAAAGCGAAATTTATA  60

Strep_263  61    AAGTGTGAAAAGGAGTGAAAACTTTCTTTTTTCTAATTAAAATAGCGGAACAATTAGAAT  120

             ||||||||||||||||||||||||| ||||||||||||||||||||||||||||||||||

PS9 Clone  61    AAGTGTGAAAAGGAGTGAAAACTTTATTTTTTCTAATTAAAATAGCGGAACAATTAGAAT  120

Strep_263  121   TGGAAGATCCGTTTACAGGAAAATATAAATAGGTGCTCGTCGCAGGCCACACGAGCGCCT  180

             ||||||||||||||||||||||||||||||||||||||||||||||||||||||||||||

PS9 Clone  121   TGGAAGATCCGTTTACAGGAAAATATAAATAGGTGCTCGTCGCAGGCCACACGAGCGCCT  180

Strep_263  181   TGAGGCGTCGCTCTGAGATCAGAGCTATGCCCGAAATTGAAAAGCCACCCGCTAAGCCAC  240

             ||||||||||||||||||||||||||||||||||||||||||||||||||||||||||||

PS9 Clone  181   TGAGGCGTCGCTCTGAGATCAGAGCTATGCCCGAAATTGAAAAGCCACCCGCTAAGCCAC  240

Strep_263  241   TGGATATTTATTATGGGAGAAATTATCATATATGATAATTTCGCAAAGTGTGCCTCATCG  300

             ||||||||||||||||||||||||||||||||||||||||||||||||||||| ||||||

PS9 Clone  241   TGGATATTTATTATGGGAGAAATTATCATATATGATAATTTCGCAAAGTGTGCTTCATCG  300

Strep_263  301   ATTTGGTTCGCCTTAAGGGTCCATGGTATAATAAACAAGACAAAATAAGAAATGGGGAAA  360

             |||||||||||||| ||||||||||||||||| || |||| |||||||||||||||||||

PS9 Clone  301   ATTTGGTTCGCCTTGAGGGTCCATGGTATAATGAATAAGATAAAATAAGAAATGGGGAAA  360

Strep_263  361   CCACATGAAACCATTTGATTACATCGTTGTGGGTGCTGGCCTTTTCGGTGCAACCTTTGC  420

             |||||||||||||||||||||||||||||||||||||||  | |||||||| ||||||||

UDP Galactopyranose

PS9 Clone  361   CCACATGAAACCATTTGATTACATCGTTGTGGGTGCTGGATTATTCGGTGCGACCTTTGC  420

Strep_263  421   GCATGAAGCAGCGACACGCGGCTATAAAGTAAAAGTGATTGAGAAAAGAAATCATATTGC  480

             ||||||||||||||||||||| ||||||||||||||||||||||| ||||||||||||||

PS9 Clone  421   GCATGAAGCAGCGACACGCGGTTATAAAGTAAAAGTGATTGAGAAGAGAAATCATATTGC  480

Strep_263  481   AGGGAATATTTATACAAAAGAAGTGGAAGGCATTCAAGTGCACGAATATGGTGCGCATAT  540

              ||||||||||||||||||||||||||||||||||||||||||||||| |||||||||||

PS9 Clone  481   GGGGAATATTTATACAAAAGAAGTGGAAGGCATTCAAGTGCACGAATACGGTGCGCATAT  540

Strep_263  541   TTTCCATACGAGCGACAAAAAGATTTGGGATTACGTGCATCAGTTTGCAACGTTTAATCG  600

             |||||| ||||||||||| ||||||||||||||||||||||||||||| || ||||||||

PS9 Clone  541   TTTCCACACGAGCGACAAGAAGATTTGGGATTACGTGCATCAGTTTGCGACATTTAATCG  600

Strep_263  601   CTATACGAATACTCCAGTTGCCAACTTCAACGGAGAAATCTACAACTTGCCCTTTAACAT  660

              |||||| |||||||||||||||||||||||||||||||||||||||||||||||||||||

PS9 Clone  601   CTATACCAATACTCCAGTTGCCAACTTCAACGGAGAAATCTACAACTTGCCCTTTAACAT  660

Strep_263  661   GAATACTTTTAATAAATTATGGGGTGTGGTGACGCCACAAGAAGCCGAAGCGAAGATCGC  720

             ||||||||| |||||||||||||||||||||||||||||||||||||||||||| || ||

PS9 Clone  661   GAATACTTTCAATAAATTATGGGGTGTGGTGACGCCACAAGAAGCCGAAGCGAAAATTGC  720

Strep_263  721   TGAGCAACGTGCGGTTCTTGGCGGTAAAGAGCCTGAAAACTTAGAAGAGCAGGCGATTTC  780

             ||| || |||||||| || || |  ||||||||||||||||||| |||||||||||||||

PS9 Clone  721   TGAACAGCGTGCGGTACTCGGGGACAAAGAGCCTGAAAACTTAGTAGAGCAGGCGATTTC  780

Strep_263  781   TCTTGTGGGAGAAGATATTTACTACAAGCTCATCAAGGGCTATACGGAGAAGCAATGGGG  840

             |||||| || ||||||||||||||||||||||||||||||||||||||||| ||||||||

PS9 Clone  781   TCTTGTTGGGGAAGATATTTACTACAAGCTCATCAAGGGCTATACGGAGAAACAATGGGG  840

Strep_263  841   ACGTTCGGCTACAGAATTACCAGCGTTTATCATCCGCCGCTTGCCGGTTCGTTATACTTA  900

             |||||||||||||||||||||||| |||||||| |||||||||||||||||||| |||||

PS9 Clone  841   ACGTTCGGCTACAGAATTACCAGCATTTATCATTCGCCGCTTGCCGGTTCGTTACACTTA  900

Strep_263  901   CGACAATAACTACTTTAACGATACGTACCAAGGGATTCCGATTGGCGGCTATACGAAGAT  960

             ||| || ||||| |||||||||||||||||||||||||||||||| ||||||||||||||

PS9 Clone  901   CGATAACAACTATTTTAACGATACGTACCAAGGGATTCCGATTGGTGGCTATACGAAGAT  960

Strep_263  961   GATTGAAGCCATGCTGGACCATGAGAATATCGAAGTTGAGTTGAATGTCGATTTCTTCGC  1020

             |||||||||||||||||||||||||||||||||||| ||||||||||| |||||||| ||

PS9 Clone  961   GATTGAAGCCATGCTGGACCATGAGAATATCGAAGTCGAGTTGAATGTTGATTTCTTTGC  1020

Strep_263  1021  GAAAAAAGACGAGTACTTAAGTAGTGGTGCCAAAATCGTCTTCACGGGAATGATTGATGA  1080

             ||||||||| ||||||||||||||||||||||||||||||||||||||||||||||||||

PS9 Clone  1021  GAAAAAAGATGAGTACTTAAGTAGTGGTGCCAAAATCGTCTTCACGGGAATGATTGATGA  1080

Strep_263  1081  GTTCTTCGATTATGAATTAGGTACATTAGAATACCGTTCGCTCCGTTTTGAAACAGAAGT  1140

             |||||||||||||||| |||| ||||||||||||||||||||||||||||||||||||||

PS9 Clone  1081  GTTCTTCGATTATGAACTAGGCACATTAGAATACCGTTCGCTCCGTTTTGAAACAGAAGT  1140

Strep_263  1141  CGTGGACGTGGAGAACTATCAAGGAAATGCGGTGGTAAACTATACAGACCGCGAAACGCC  1200

             ||||||||||||||||||||||||||||||||| ||||||||||||||||||||||||||

PS9 Clone  1141  CGTGGACGTGGAGAACTATCAAGGAAATGCGGTAGTAAACTATACAGACCGCGAAACGCC  1200

Strep_263  1201  ATACACTCGTATCATCGAGCATAAGCATTTCGAGTTTGGCACTCAGCCGAAAACGGTAAT  1260

             |||||||||||||||||||||||||||||||||||||||||| ||||| |||||||| ||

PS9 Clone  1201  ATACACTCGTATCATCGAGCATAAGCATTTCGAGTTTGGCACGCAGCCCAAAACGGTGAT  1260

Strep_263  1261  TACGCGTGAGTATCCTGCCGATTGGAAAGTGGGAGATGAGCCTTATTACCCAGTGAATAA  1320

             |||||||||||||||||||||||||||||||||||| ||||||||||| |||||||||||

PS9 Clone  1261  TACGCGTGAGTATCCTGCCGATTGGAAAGTGGGAGACGAGCCTTATTATCCAGTGAATAA  1320

Strep_263  1321  TAAAGTGAACAACGACTTGTACGCACAGTATAAGAAATTAGCGCAGACGGTTCCGCAAGT  1380

             |||||| ||||||||||||||||| |||||||||||||||||||||||||||||||||||

PS9 Clone  1321  TAAAGTCAACAACGACTTGTACGCGCAGTATAAGAAATTAGCGCAGACGGTTCCGCAAGT  1380

Strep_263  1381  GATTTTCGGAGGACGCCTTGGACAGTATCGTTATTACGATATGCATCAAGTTATCGCCGC  1440

             ||||||||||||||||||||||||||||||||||||||||||||||||||| ||||||||

PS9 Clone  1381  GATTTTCGGAGGACGCCTTGGACAGTATCGTTATTACGATATGCATCAAGTCATCGCCGC  1440

Strep_263  1441  TGCCCTAGAAACGGTT-AAACATGAATTTGAATAAATGCAAAAACACCCTATTTGGTCAT  1499

              || |||||||| ||  | | | |||||    |||| |||| |||||| |||||||||||

PS9 Clone  1441  AGCACTAGAAACTGTCGATAAA-GAATTCCGTTAAACGCAAGAACACCATATTTGGTCAT  1499

Strep_263  1500  GGCCAAATAGGGTCTTATTT  1519

             ||||||||| ||| || |||

PS9 Clone  1500  GGCCAAATATGGTGTT-TTT  1518

Next the inversion;

Strep_263  1526  GATTCCTTCGAATCCTAGCGGATTTTATCATAACTACAATAATGGGGTACCGGTTCGAGC  1585

             ||| ||| ||||||||||||||||||||||||||||||||||||||||||||||||||||

PS9 Clone  1789  GATACCTACGAATCCTAGCGGATTTTATCATAACTACAATAATGGGGTACCGGTTCGAGC  1730

Strep_263  1586  CTGTAGTCTCTCACCTTTAAAGCTTCAAAAAGGGCGTAAGGAATAAATCCCTA-CGCC--  1642

             || |||||||||||||||||||| |||||   || |||||||||||||  ||| | ||

PS9 Clone  1729  CTATAGTCTCTCACCTTTAAAGCCTCAAAGCTGGAGTAAGGAATAAATTACTAACTCCAG  1670

Strep_263  1643  CTTTAATTCATCTTTAATACGATTCCCCATTATTGTGGCTATGAAATCCGCAAGATTCTT  1702

             ||||  |||   |||||||| ||||||||||||||||| |||||||||||||||||||||

PS9 Clone  1669  CTTTT-TTCGCATTTAATACCATTCCCCATTATTGTGGTTATGAAATCCGCAAGATTCTT  1611

Strep_263  1703  CGG  1705

              |||

PS9 Clone  1610  CGG  1608

The sequence then continues on…

Strep_263  1751  ATTAAGCATTCACTGTATCTAGTTCTTCGCCGATAGCTTCAAGACGAGCTACAACTTCTT  1810

         ||||||||||||||||||||||||||||||||||||||| ||||||||  ||||| ||||

PS9 Clone  1822  ATTAAGCATTCACTGTATCTAGTTCTTCGCCGATAGCTTGAAGACGAGAGACAACCTCTT  1881

Strep_263  1811  CAAGAGACAAGTTGTGTTCTTTCACGTAGCGGTTGCGTGGATGAACACGACATTCATGGC  1870

             ||||||| | ||||||||||||||| |||||||||||||| |||||||||||||||||||

PS9 Clone  1882  CAAGAGATAGGTTGTGTTCTTTCACATAGCGGTTGCGTGGGTGAACACGACATTCATGGC  1941

Strep_263  1871  TGCAACCACGTAAGTATTTGTCTTCGTTCTCTTCTGAAGCTAAGATGCGACGGTTACATT  1930

             |||||||||||||||||||||||||||||||||||||||||||||| |||||||||||||

PS9 Clone  1942  TGCAACCACGTAAGTATTTGTCTTCGTTCTCTTCTGAAGCTAAGATACGACGGTTACATT  2001

Strep_263  1931  CTGGGTTTCCACAGTTTACGTAACGTTCGCAAGGTGTACCATCGAACCAGTCTTTCCCGA  1990

              |||||||||||||||||||||||||||||||||||||||||||||||||||||||||| |

PS9 Clone  2002  CTGGGTTTCCACAGTTTACGTAACGTTCGCAAGGTGTACCATCGAACCAGTCTTTCCCAA  2061

Strep_263  1991  TAACAACTGGGTCTACGTGGTTGATGTCAACAGCGATACGCTCGTCGAATACGTACATTT  2050

             | || ||||| ||||||||||||||||||||||||||||| ||||| |||||||||||||

PS9 Clone  2062  TTACTACTGGATCTACGTGGTTGATGTCAACAGCGATACGTTCGTCAAATACGTACATTT  2121

Strep_263  2051  TTCCATCCCAAAGTTCGCCTTGAACTTCTGGGTCTTTACCGTAAGTTGCGATACCACCGT  2110

             |||||||||| | |||||||||||||||||||||||| ||||||||||||||||| ||||

PS9 Clone  2122  TTCCATCCCATAATTCGCCTTGAACTTCTGGGTCTTTCCCGTAAGTTGCGATACCGCCGT  2181

Strep_263  2111  GTAATTGACCGACATCTTTGTAACCTTCACGAACCATCCAGCCAGAGAATTTCTCACAGC  2170

             |||||||||||||||||||||| |||||||||||||||||||||||||||||||||||||

PS9 Clone  2182  GTAATTGACCGACATCTTTGTAGCCTTCACGAACCATCCAGCCAGAGAATTTCTCACAGC  2241

Strep_263  2171  GAACGCCACCTGTACAGTAAACAACGACACGTTTGTCCATGAATTTCTCTTTGTTATCGC  2230

             ||||||||||||||||||||||||||||||||||||||||||||||||||||||||||||

PS9 Clone  2242  GAACGCCACCTGTACAGTAAACAACGACACGTTTGTCCATGAATTTCTCTTTGTTATCGC  2301

Strep_263  2231  GAACCCATTGTGGTAATTCACGGAAGTTGCGAATGTCAGGACGGATAGCACCGCGGAAGT  2290

             |||||||||||||||||||||||||||||||||||||||||||||| |||||||||||||

PS9 Clone  2302  GAACCCATTGTGGTAATTCACGGAAGTTGCGAATGTCAGGACGGATGGCACCGCGGAAGT  2361

Strep_263  2291  GACCTAGATCGTACTCATAGTCGTTACGAGTGTCTAGGACAACAGTGTTTTCGTCAAGGA  2350

             ||||||| |||||||| |||||||||||||||||||| |||||||||||||||||||| |

PS9 Clone  2362  GACCTAGGTCGTACTCGTAGTCGTTACGAGTGTCTAGTACAACAGTGTTTTCGTCAAGTA  2421

Strep_263  2351  GTGCTTCCTTGAATTCTTTTGGTGAAAGGTAAGCACCTGTTGTTTCAAGCGGGTTAATAT  2410

             | ||||| || || |||| ||| ||||||||||| |||||||||||||| ||||||||||

PS9 Clone  2422  GGGCTTCTTTAAACTCTTGTGGAGAAAGGTAAGCCCCTGTTGTTTCAAGTGGGTTAATAT  2481

Strep_263  2411  CGTTATCGAAGTTGTTATCCTCTAAGCCAAGGTGTACGATTTCTTTTTTGTAACGAACGA  2470

             |||| ||||||| |||||| ||||| ||||||||||||||||||||||||||||||||||

PS9 Clone  2482  CGTTGTCGAAGTCGTTATCTTCTAAACCAAGGTGTACGATTTCTTTTTTGTAACGAACGA  2541

Strep_263  2471  ACATCTTCTTGAAGGCTTGTTCGCTTTCTTCATCGATTTTAAACCATAAGTCTTCCATGC  2530

             ||||||||||||||||||| ||||||||||| |||||||||||||||||||||||||| |

PS9 Clone  2542  ACATCTTCTTGAAGGCTTGCTCGCTTTCTTCGTCGATTTTAAACCATAAGTCTTCCATCC  2601

Strep_263  2531  CAGGAAGAGAGTGAACATAGTCCATATATTTTTGAGTTGTTTCGTAATCACCAGATACAG  2590

             | |||||||||||||||||||||||||||||||||||||||||||||||||||||||| |

PS9 Clone  2602  CTGGAAGAGAGTGAACATAGTCCATATATTTTTGAGTTGTTTCGTAATCACCAGATACCG  2661

Strep_263  2591  TTCCGTTGATTCCTTCGTCAGCGACAAGGATACGTCCTTTTAATCCGATTGATTTGCAGA  2650

             ||||||| ||||||||||||||||||||||||||||||||||||||||||||||||||||

PS9 Clone  2662  TTCCGTTAATTCCTTCGTCAGCGACAAGGATACGTCCTTTTAATCCGATTGATTTGCAGA  2721

Strep_263  2651  ATGCTAAATGATCTGCCGCAAATTGTTCCGCATTTTCGATTGGAACATATTTATAGTAAA  2710

             ||||||||||||||||||| ||||||||||||||||||||||||||||||||||||||||

PS9 Clone  2722  ATGCTAAATGATCTGCCGCGAATTGTTCCGCATTTTCGATTGGAACATATTTATAGTAAA  2781

Strep_263  2711  GTAAAACACGAATGTCTTTTGCCATATTGTAAATCTTTCATCCTTTTCTAATTTGTAATG  2770

             ||||||||||||||||||||||||||||||||||||||||||||||||||||||||||||

Sulfurtransferase

PS9 Clone  2782  GTAAAACACGAATGTCTTTTGCCATATTGTAAATCTTTCATCCTTTTCTAATTTGTAATG  2841

Strep_263  2771  ATTCTATTGTAACCGTATTGAGAACCATTATCAATGAATTCGATTGTGAAAAACTTCGAA  2830

             ||||||||||||| ||||||||||  ||||||||||||||||||||||||||| ||||||

PS9 Clone  2842  ATTCTATTGTAACTGTATTGAGAATGATTATCAATGAATTCGATTGTGAAAAAGTTCGAA  2901

Strep_263  2831  AGGTATTGACATGACGGCATTTTAGAGTATAATTCGAAGTATAACGTTAAATTTATCAGA  2890

             ||||||||||||||||| ||||||  |||||||||  | |||||||||||||||||||||

PS9 Clone  2902  AGGTATTGACATGACGGTATTTTACGGTATAATTCAGA-TATAACGTTAAATTTATCAGA  2960

Strep_263  2891  TTGGAGGAGAAATCAATGAACAACTTAAACGTAAAACAAGTACATGATTTGAGAAACCTT  2950

             |||||||||||||||||||||||||||||||||||||||||||| |||||||||||||||

PS9 Clone  2961  TTGGAGGAGAAATCAATGAACAACTTAAACGTAAAACAAGTACAAGATTTGAGAAACCTT  3020

Strep_263  2951  CCAATTTTGGTGAATGAAGTATAGCGCATTTTTATAGATAAATTTAAGTGAGCCGTTACT  3010

             ||||||||||||||||||||||||||||||||||||||||| ||||||||||||||||||

PS9 Clone  3021  CCAATTTTGGTGAATGAAGTATAGCGCATTTTTATAGATAAGTTTAAGTGAGCCGTTACT  3080

Strep_263  3011  TTTTCCATGCGTGGGGAAGGTGACGGCTTTTTTGATGGAGGGAAACGATGAACGATTTAT  3070

*tet*A(60)

             ||||||||||||||||||||||||||||||||||||||||| ||||||||||||||||||

PS9 Clone  3081  TTTTCCATGCGTGGGGAAGGTGACGGCTTTTTTGATGGAGGAAAACGATGAACGATTTAT  3140

Strep_263  3071  TAAAAATAGTGATAGGCTTCATAAAAAAACATCCGATGCGCTACCTTGTTAGTTTTATTT  3130

             ||||| |  | ||    || ||||| ||||||||||||||||||||||||||||||||||

PS9 Clone  3141  TAAAAGTCATTATTAATTTTATAAAGAAACATCCGATGCGCTACCTTGTTAGTTTTATTT  3200

Strep_263  3131  TGATGATCGGAAGTAGTATTGCGGCGGTATACCCAGCGCGTATTATCGGACAAGTCGTCG  3190

             |||||||||||||||||||||||||||| |||||||||||||||||||||||||| || |

PS9 Clone  3201  TGATGATCGGAAGTAGTATTGCGGCGGTGTACCCAGCGCGTATTATCGGACAAGTTGTTG  3260

Strep_263  3191  ATAAAATCGTGGCGAGTGAACTGAATGCCGAGTGGCTTGGGACACAGCTCGTGATTTTAG  3250

             |||||||||| ||||| ||||||||||||||||||||||||||||| |||||||||||||

PS9 Clone  3261  ATAAAATCGTAGCGAGCGAACTGAATGCCGAGTGGCTTGGGACACAACTCGTGATTTTAG  3320

Strep_263  3251  TCGGGATTATTCTTGTGGCCTATATTACGGAGAGTATTTGGACATATTTTATTTTTATTG  3310

             ||||||||||||||||||| ||||||||||||||||||||||||||||||||||||||||

PS9 Clone  3321  TCGGGATTATTCTTGTGGCGTATATTACGGAGAGTATTTGGACATATTTTATTTTTATTG  3380

Strep_263  3311  GGTATTATGAAATTCAAAAAGAATTACGTGTGAAGTTACTACGTAATAATTTACGGAAGA  3370

             ||||||||||||||||||||||||||||||||||||||||||||||||||||||||||||

PS9 Clone  3381  GGTATTATGAAATTCAAAAAGAATTACGTGTGAAGTTACTACGTAATAATTTACGGAAGA  3440

Strep_263  3371  AAATTCCGTTTTATGCGCATTTTAGAACGGGCGATATTATTACGCGTAGCAGTGAAGACG  3430

             |||||||||||||||||||||||||||||||||| |||||||||||||||||||||||||

PS9 Clone  3441  AAATTCCGTTTTATGCGCATTTTAGAACGGGCGAAATTATTACGCGTAGCAGTGAAGACG  3500

Strep_263  3431  TTACAACGATTGGCGATATGATGGGGTTTGGGATGTTTGCATTGATGAACTCTACATTGC  3490

             ||||||||||||||||||||||||||||||||||||||||||||||||||||||||||||

PS9 Clone  3501  TTACAACGATTGGCGATATGATGGGGTTTGGGATGTTTGCATTGATGAACTCTACATTGC  3560

Strep_263  3491  TGATGAGCGTATCGATTTATATGATGGTCACAACGATTTCGTTGCCACTGACCATCGCAG  3550

             |||||||||||||||||||||||||||||||||||||||| |||||||||||||||||||

PS9 Clone  3561  TGATGAGCGTATCGATTTATATGATGGTCACAACGATTTCATTGCCACTGACCATCGCAG  3620

Strep_263  3551  CGATTTTGCCACTGCCATTCCTTTCGTATCTTGTATATAAATGGGGATTCGATTTAGAAG  3610

             ||||||||||||||||| ||||||||||||||||||||||||||||||||||||||||||

PS9 Clone  3621  CGATTTTGCCACTGCCAATCCTTTCGTATCTTGTATATAAATGGGGATTCGATTTAGAAG  3680

Strep_263  3611  AAGAGTACAACAAGGCGCAAAATGCAGTTTCACAATTAAATAATGAAGTGCTTGAGATGG  3670

             |||||||||||||||||||||||||||||||||||||||||||||||||||||||||||

PS9 Clone  3681  AAGAGTACAACAAGGCGCAAAATGCAGTTTCACAATTAAATAATGAAGTGCTTGAGATGA  3740

Strep_263  3671  TTGACGGGACGTATGTGATTCGTGCTTACGGGCAAGAAGATGCGATGATGGATGAGTTCA  3730

             ||||||||||||||||||||||||||||||||||||||||||||||||||||||||||||

PS9 Clone  3741  TTGACGGGACGTATGTGATTCGTGCTTACGGGCAAGAAGATGCGATGATGGATGAGTTCA  3800

Strep_263  3731  GGGCGAAAACGAAAAAGGCCATGAAACAAAATATTATCGTGTCTGAAATTGAATCGCGCT  3790

             ||||||||||||||||||||||||||||||||||||||||| ||||||||||||||||||

PS9 Clone  3801  GGGCGAAAACGAAAAAGGCCATGAAACAAAATATTATCGTGACTGAAATTGAATCGCGCT  3860

Strep_263  3791  TTATTCCACTGGCGCAATTATTTATGATGATTAGCTTTACCATTGCCCTTCTATATGGTG  3850

             |||||||||||||||||||||||||||||||||||||||||||||||||| | || ||||

PS9 Clone  3861  TTATTCCACTGGCGCAATTATTTATGATGATTAGCTTTACCATTGCCCTTTTCTACGGTG  3920

Strep_263  3851  GATATTTGGTATCGACGGGGGCCATTCTAGTCGGGGATGTCATTGCCTTCCAAGTTTATA  3910

             | ||| | |||||||| ||||| |||||||||||||||||||||||||||||||| ||||

PS9 Clone  3921  GGTATCTAGTATCGACTGGGGCTATTCTAGTCGGGGATGTCATTGCCTTCCAAGTCTATA  3980

Strep_263  3911  TGGGGGCGATTATGTGGCCGATGTTTATGATTGGCGATATTATTACGAACTATAAACGCG  3970

             ||||||||||||||||||||||||||||||||||||||||||||||||||||||||||||

PS9 Clone  3981  TGGGGGCGATTATGTGGCCGATGTTTATGATTGGCGATATTATTACGAACTATAAACGCG  4040

Strep_263  3971  GAAAAGTGGCAACGGAGCGTATTAATGAAGTGTTGAAACATGATGATGAAATTGAACGTG  4030

             |||||||||| |||||||||||||||||||||||||||||||| |||||||||||||| |

PS9 Clone  4041  GAAAAGTGGCGACGGAGCGTATTAATGAAGTGTTGAAACATGACGATGAAATTGAACGCG  4100

Strep_263  4031  GCGGTACAAAAGCGCTCGAGACAATCGAATCCATTGAGTTTAAGGACTTCAATTTTACGT  4090

             ||||||||||| |||||||||| || |||||||||||||||||||||||| |||||| ||

PS9 Clone  4101  GCGGTACAAAAACGCTCGAGACGATTGAATCCATTGAGTTTAAGGACTTCCATTTTATGT  4160

Strep_263  4091  ATCCAGGCGAAGAGGTGCCATTATTAAAAGAGATTAACCTTACATTAAATAAAGGCGAGA  4150

             |||||||||||||||  |||||||||||||||||||||||||| |||  |||||||||||

PS9 Clone  4161  ATCCAGGCGAAGAGGCACCATTATTAAAAGAGATTAACCTTACGTTACGTAAAGGCGAGA  4220

Strep_263  4151  CGCTTGGGATTGTAGGAAAAACAGGTTCTGGGAAGACAACGCTCTTGATGCAATTATTAC  4210

             ||||||| || || |||||||| |||||||||||||| ||||||||||||||||||||||

PS9 Clone  4221  CGCTTGGAATCGTTGGAAAAACGGGTTCTGGGAAGACGACGCTCTTGATGCAATTATTAC  4280

Strep_263  4211  ATCAATTTCCATACCGAGGCGAGAAACTGCTCATTAATGGAGAGCCATTGATTGATTACG  4270

             |||||||||| |||||||| ||||| ||||||||||| ||||||||||||||||||||||

PS9 Clone  4281  ATCAATTTCCGTACCGAGGAGAGAAGCTGCTCATTAACGGAGAGCCATTGATTGATTACG  4340

Strep_263  4271  ATTCTCAAATGGTGGCGGGACATTTAGCCTACGTTCCACAAGAACACACGCTTTTCTCAC  4330

             |  |||||  |||||| || ||| ||||||| || |||||||||||||| ||||||||||

PS9 Clone  4341  ACACTCAATCGGTGGCAGGGCATCTAGCCTATGTGCCACAAGAACACACCCTTTTCTCAC  4400

Strep_263  4331  GCACGATTCGTGAGAATATGTTATTCGGGAAAGAAGATGCGACCGATGAGGAAATTTGGG  4390

             |||||||||| ||||||||||||||||| ||||| ||||| || ||||| ||||||||||

PS9 Clone  4401  GCACGATTCGCGAGAATATGTTATTCGGAAAAGAGGATGCAACGGATGATGAAATTTGGG  4460

Strep_263  4391  AAGCGTTGACGTTGGCGTCCTTTGATGGAGACGTGAAACGCATGCCACAGCAGCTCGATA  4450

             ||||||||||| | || || ||||| |||||||||||||| |||||| |  |||||||||

PS9 Clone  4461  AAGCGTTGACGCTAGCCTCTTTTGAAGGAGACGTGAAACGAATGCCAGACGAGCTCGATA  4520

Strep_263  4451  CGATGGTCGGAGAAAAAGGAGTATCACTTAGTGGTGGCCAAAAACAACGGCTATCGATTG  4510

             ||||||||||||||||||| ||||| || ||||| || |||||||||||  |||||||||

PS9 Clone  4521  CGATGGTCGGAGAAAAAGGGGTATCGCTCAGTGGAGGTCAAAAACAACGCTTATCGATTG  4580

Strep_263  4511  CGCGTGCCTTCTTACGCAACCGTGAATGTTTGATTTTAGATGATGCGTTATCCGCAGTTG  4570

             | ||||| |||||||||||||||||||| || ||||| |||||||||||||| |||||||

PS9 Clone  4581  CTCGTGCTTTCTTACGCAACCGTGAATGCTTAATTTTGGATGATGCGTTATCTGCAGTTG  4640

*L. buchneri* CD034 regions underlined

Strep_263  4571  ATGCGAAAACGGAACGCGAAATTATCTCGCACTTGCAAGAAGAGCGCGGGGGCTGTATGA  4630

             |||||||||||||| | ||||||||||||||||||||| |||| ||||| || |||||||

PS9 Clone  4641  ATGCGAAAACGGAAAGGGAAATTATCTCGCACTTGCAACAAGAACGCGGAGGTTGTATGA  4700

Strep_263  4631  ATATTATTTCTGCACACCGACTCTCTGCGATTCGTCATGCCGATGAGATTATTGTGATGA  4690

             |||| |||||||| ||| |||| ||||| ||||||||||| ||||| |||||||||||||

PS9 Clone  4701  ATATCATTTCTGCGCACAGACTTTCTGCAATTCGTCATGCGGATGAAATTATTGTGATGA  4760

Strep_263  4691  ATGAAGGACGCATTAGCGAGCGCGGCACCCACGAGGAGTTACTCGCACAACGTGGTTGGT  4750

             |||||||||| ||||| ||| | || |||||||||||| | |||| |||||| || ||||

PS9 Clone  4761  ATGAAGGACGTATTAGTGAGAGGGGTACCCACGAGGAGCTGCTCGAACAACGAGGATGGT  4820

Strep_263  4751  ACTATGAACAGTACCTCATTCAAGAGATAGAGGAGGAGATCGAATGAAAACGATGAAGCG  4810

             ||||||||||||| ||||  ||||| || |||||||| ||||||||| ||||||||||||

PS9 Clone  4821  ACTATGAACAGTATCTCACACAAGAAATGGAGGAGGAAATCGAATGAGAACGATGAAGCG  4880

*tet*B(60)

Strep_263  4811  ATTATTAAGCTACCTCCGCTATGAGAAAAAAGGAGTTCTGATTGGACTCGTCTGCTTGTT  4870

             |||||||||||||||||| |||||||||||||||||||||||||||||| ||||| ||||

PS9 Clone  4881  ATTATTAAGCTACCTCCGTTATGAGAAAAAAGGAGTTCTGATTGGACTCTTCTGCCTGTT  4940

Strep_263  4871  GATTTCAACTATCGCAACTTTAACAGGACCTCTTGTGGCAAAATATATGATTGATAATGT  4930

               | |||||    ||||| |||||||| || || || || ||| | || || ||||||||

PS9 Clone  4941  ACTCTCAACAGGAGCAACCTTAACAGGGCCACTCGTTGCGAAACACATTATCGATAATGT  5000

Strep_263  4931  GATTACACCGATGGGACAATCGCATGTCTTCGATGCTGGAAGTGTACTCCTATGGGTGGG  4990

             |||||| |||||||| ||  ||||||  ||| | || ||  |  ||||  ||||||| ||

PS9 Clone  5001  GATTACTCCGATGGGGCAGGCGCATGATTTCAAAGCAGGTGGCCTACTTTTATGGGTTGG  5060

Strep_263  4991  TATTTACGTGGCAGTGAACTTAATTGGAGCGGCTGGTGGATATTTAAACCGTTTATATAT  5050

             |||||||||| ||||||||||| | ||||  ||||| |  |||||||||||| |||||||

PS9 Clone  5061  TATTTACGTGACAGTGAACTTAGTGGGAGTTGCTGGGGCCTATTTAAACCGTGTATATAT  5120

Strep_263  5051  GAAGACTCTCTCGAACCGCGTGGCCAAACGCATTCGTGACGAAGTATTCGAACATGTACA  5110

             |||||||||||| |||||  | || |||||||||||||||||||| ||||| ||||||||

PS9 Clone  5121  GAAGACTCTCTCAAACCGTATTGCAAAACGCATTCGTGACGAAGTGTTCGAGCATGTACA  5180

Strep_263  5111  AACCTTGCCAGTATCGTATTTCGACCATTTACCTGCCGGAAAAGTGGTTTCTAGAATTAC  5170

             ||||||||| |||||||| |||||||||||||| || || ||||||||||||||||||||

PS9 Clone  5181  AACCTTGCCGGTATCGTACTTCGACCATTTACCAGCTGGGAAAGTGGTTTCTAGAATTAC  5240

Strep_263  5171  GAGTGATACGGAATCGGTGCGTGCAAACTTTTATGTCAATGGGATTTCGACGCTCCTTAG  5230

              |||||||| ||||| |||||||||||||| || || | |||||||||||||||  | ||

PS9 Clone  5241  AAGTGATACAGAATCAGTGCGTGCAAACTTCTACGTGAGTGGGATTTCGACGCTTTTCAG  5300

Strep_263  5231  TACCGTCGTGATGCTCATTGGGGTATACACAACGATTTTCTTATTAAATGCGACGCTTGG  5290

             |||  | |||||| |  | || || || | ||| |||||||||||||| || ||||| ||

PS9 Clone  5301  TACGATTGTGATGTTGGTAGGTGTTTATATAACCATTTTCTTATTAAACGCAACGCTAGG  5360

Strep_263  5291  ATTCGTGTTATTATTCCTAGTTCCGGTGATGATGCTATGGCAGCGAACCGTTGCCGTGAT  5350

             | |||| ||||||||||||||||| |||||||| ||||||||| ||||||||||   ||

PS9 Clone  5361  ACTCGTATTATTATTCCTAGTTCCTGTGATGATTCTATGGCAGAGAACCGTTGCTACGAA  5420

Strep_263  5351  GCAGAAAAAATGCTATACAGAGAGTCGTGAACTGTACAGCCAGTTGAGCGGACAGTTAAA  5410

              ||||||||||| |||| | || | ||||||||| || || |||||||| ||||| |||||

PS9 Clone  5421  GCAGAAAAAATACTATTCCGAAAATCGTGAACTCTATAGTCAGTTGAGTGGACAATTAAA  5480

Strep_263  5411  TGAAAGCATTCAAGGGGCTGGTATCGTTCAAGCCTTCCAACAAGAAGAAAAAATTGTCGC  5470

              |||||||||||||| || || ||||||||||||||||| ||||||||||||||||| ||

PS9 Clone  5481  CGAAAGCATTCAAGGAGCAGGCATCGTTCAAGCCTTCCAGCAAGAAGAAAAAATTGTTGC  5540

Strep_263  5471  GGAATATGATGAGACGGCTACGTCTTGGGTAGAGGTTGGTCGCAAGGAATTAATTCTCGA  5530

              ||||||||||  ||||| || ||||||||||| |||||||| |||||||||||||| ||

PS9 Clone  5541  AGAATATGATGCCACGGCAACTTCTTGGGTAGAAGTTGGTCGTAAGGAATTAATTCTTGA  5600

Strep_263  5531  TTCTTACTTCTCGTGGAGTTTAGTGAGCTTACTTCGTAACTTTACGCTATTTGGAATTGT  5590

              || ||||||||||||||| | ||  || | ||||| ||| |||| |  |||||| || |

PS9 Clone  5601  GTCGTACTTCTCGTGGAGTCTTGTCGGCATGCTTCGAAACATTACTCATTTTGGAGTTAT  5660

Strep_263  5591  GTATTACTTCGGAATGCAATTTATCGGAGGAACACTTGGAATTTCAGCGGGGCTCCTCTA  5650

              ||||| ||| | ||||| |||||||| |||||||| || |||||||| || ||  | ||

PS9 Clone  5661  CTATTATTTCAGTATGCAGTTTATCGGTGGAACACTCGGGATTTCAGCAGGTCTTTTATA  5720

Strep_263  5651  TGCGTTTATTGATTATATCAACCGCGTTTATGAGCCGATTCAAACCTTTATGAATGTCGT  5710

             ||| |||||||| || || || ||  | ||||||||||||||||| ||||||||||||||

PS9 Clone  5721  TGCATTTATTGACTACATTAATCGTATATATGAGCCGATTCAAACTTTTATGAATGTCGT  5780

Strep_263  5711  GTCTGGTTTCCAACAATCGATGGCTGCGGGTGACCGCGTGTTTGAATTGATGGATACGCC  5770

             |||||| ||||| ||||| |||||||| |||||||| ||||||||| | |||||||| ||

PS9 Clone  5781  GTCTGGCTTCCAGCAATCAATGGCTGCTGGTGACCGTGTGTTTGAACTAATGGATACACC  5840

Strep_263  5771  AAGCGAGGAAAGTGGCGAAGAGCTCTTCACATTCGACGAAGGACGTATCGAATTTAAGGA  5830

              |||||||||   || |||||||||||||| || || |||||| ||||||||||||| ||

PS9 Clone  5841  GAGCGAGGAATCGGGAGAAGAGCTCTTCACGTTTGATGAAGGATGTATCGAATTTAAAGA  5900

Strep_263  5831  TGTGAGCTTCGAGTATACGCCAGGAGTACCGGTGTTGAAACACTTGAATTTCACGGTAGA  5890

             |||||| || ||||| ||  | ||||| || |||||||||||||||||||||||||||||

PS9 Clone  5901  TGTGAGTTTTGAGTACACAGCGGGAGTTCCTGTGTTGAAACACTTGAATTTCACGGTAGA  5960

Strep_263  5891  GCCTGGGCAGACGGTGGCCTTTGTCGGTCATACCGGTTCAGGAAAATCTTCTATTATGAA  5950

              || || |||||||| || |||||||| ||||| |||||||| ||||| || ||||||||

PS9 Clone  5961  ACCAGGACAGACGGTTGCTTTTGTCGGACATACTGGTTCAGGGAAATCATCCATTATGAA  6020

Strep_263  5951  CTTGCTCTTTCGATTCTATGACCCAACGAGTGGAGCGATCTTGATTGATGGCAAAAACAC  6010

             ||| || ||||| ||||| |||||||||||||| || ||||| || ||||||||||||||

PS9 Clone  6021  CTTACTGTTTCGCTTCTACGACCCAACGAGTGGTGCTATCTTCATCGATGGCAAAAACAC  6080

Strep_263  6011  GCGTGACTTTAATAGACGTAGTGTTCGAAGCGAGATGGGGATTGTGCTGCAAGATCCATA  6070

              || |||||||| |||||||||||||||||||||||||| || || || |||||||| ||

PS9 Clone  6081  ACGCGACTTTAACAGACGTAGTGTTCGAAGCGAGATGGGAATCGTTCTTCAAGATCCGTA  6140

Strep_263  6071  CCTCTTTACAGGAACGATTGCTTCTAATGTAGGGCTTAACAATGAATCGATTGAGACTGA  6130

             |||||||||||||||||||||||| || |||||||| |||||||||||||||||| ||||

PS9 Clone  6141  CCTCTTTACAGGAACGATTGCTTCAAACGTAGGGCTCAACAATGAATCGATTGAGCCTGA  6200

Strep_263  6131  GACGATAAGAGATGCGCTTATTAAAGTAGGCGGAGGTCATCTACTCACGAAGAGTGACAA  6190

             |||||||| ||| ||| |||||||||| || ||||| |||||||| ||||||||||||||

PS9 Clone  6201  GACGATAAAAGAGGCGATTATTAAAGTGGGTGGAGGACATCTACTTACGAAGAGTGACAA  6260

Strep_263  6191  GGGCTTGGACTACGAGGTAAAAGAAAAAGGGATGGACTTCTCTTCAGGGGAACGCCAACT  6250

             |||||||||||||||||| ||||||||||| ||||| ||||||||||| |||||||||||

PS9 Clone  6261  GGGCTTGGACTACGAGGTGAAAGAAAAAGGAATGGATTTCTCTTCAGGAGAACGCCAACT  6320

Strep_263  6251  CATTTCATTTGCTCGTGCGATTGTCTTTGATCCGAAAATCTTGATTTTAGATGAAGCAAC  6310

              |||||||||||||||||||| |||||||| ||||||||||| |||||||| || |||||

PS9 Clone  6321  GATTTCATTTGCTCGTGCGATCGTCTTTGACCCGAAAATCTTAATTTTAGACGAGGCAAC  6380

Strep_263  6311  TTCGCATATTGATACCGAGACGGAAGAGATTATTCAAAACGCGATTAATGTCGTAAAAGA  6370

             ||||||||| ||||||||||| |||||||||||||| || || ||||||||||| |||||

PS9 Clone  6381  TTCGCATATCGATACCGAGACAGAAGAGATTATTCAGAATGCAATTAATGTCGTCAAAGA  6440

Strep_263  6371  AGGGCGTACAACCTTTATGATTGCGCACCGACTTTCAACGATTGCTCATGCCGACCAAAT  6430

             |||||||||||||||||||||||| ||||||||||| || ||||||||||| ||||||||

PS9 Clone  6441  AGGGCGTACAACCTTTATGATTGCTCACCGACTTTCCACAATTGCTCATGCTGACCAAAT  6500

Strep_263  6431  CTTTGTGTTGGATAAAGGCGAAATTGTGGAACGCGGAACGCATGATGAATTACTTCAACT  6490

              ||||||||||||||||| |||||||| ||||| ||||||||||||||||| ||||||||

PS9 Clone  6501  TTTTGTGTTGGATAAAGGAGAAATTGTAGAACGTGGAACGCATGATGAATTGCTTCAACT  6560

Strep_263  6491  GCAAGGGCAATATGCCGAAATGGTGGCTCTTCAAAAAGGCTAATCCTA-----T---AGA  6542

             |||||| ||||||||||||||||| || || |||||||| ||||  ||     |   |||

PS9 Clone  6561  GCAAGGTCAATATGCCGAAATGGTCGCCCTACAAAAAGGATAATTTTAAAAAATACAAGA  6620

Strep_263  6543  CTCTGTTTCCTTTCGAAACGGAGTCTTTTTTGATGCGT-GCGGTAAACAGAATTTTTAT-  6600

              | ||||||||||| ||||| | ||||||| |  | ||| |||||||||   |||||| |

PS9 Clone  6621  CGCTGTTTCCTTT-GAAACAGTGTCTTTTCTT-T-CGTAGCGGTAAACTTTATTTTTTTA  6677

Strep_263  6601  -TG-TTTTTCAAGGGAGTTCATGGTGCATAAAGCCGCCTAGTTTGTTACAATGTGTAGGA  6658

              || |||||||||||| |||||||||||| | ||| | ||||||||||||||||||||||

PS9 Clone  6678  GTGTTTTTTCAAGGGAATTCATGGTGCATTACGCCTCTTAGTTTGTTACAATGTGTAGGA  6737

Strep_263  6659  AACAGATTGGGGAGAGA-GTTATGATTACAAAAGAAGTTATTGAACGCGCTACGGCATAT  6717

             ||| ||||| |||||||   ||||||||| ||||||||||||||||| || ||  |  |

Hydrolase

PS9 Clone  6738  AACTGATTGAGGAGAGAACGTATGATTACGAAAGAAGTTATTGAACGTGCAACAACGCAC  6797

Strep_263  6718  CGCCATCATTTACATATGTATCCAGAAGTATCTGGAACGGAAGTCGAAACGACTCGTTAT  6777

             ||||  ||||||||||||||||||||||||||||||   ||||| ||||| || || |||

PS9 Clone  6798  CGCCGCCATTTACATATGTATCCAGAAGTATCTGGAGAAGAAGTTGAAACAACGCGCTAT  6857

Strep_263  6778  ATCCGCGAAGCCCTAGAAGCAATGGGGCTTACATGCTGGAATTTAAAGTCTAAAACTGGA  6837

             ||||| |||||||||||||| ||||| ||||| |||||||||||||||||||| || |||

PS9 Clone  6858  ATCCGAGAAGCCCTAGAAGCGATGGGACTTACTTGCTGGAATTTAAAGTCTAAGACGGGA  6917

Strep_263  6838  GTCGTGGCAGAAATCGGAAACGACAATGGACCAACATTAGCCTTACGTGCCGACATCGAT  6897

             || || |||||| |||||||||   | |||||||||||||||||||||||||||||||||

PS9 Clone  6918  GTGGTTGCAGAACTCGGAAACGGTGAGGGACCAACATTAGCCTTACGTGCCGACATCGAT  6977

Strep_263  6898  GCCTTACCGATTGTGGAACAAACAGGATTAGACTACGCTTCAAAAAATGAAGGAGCGATG  6957

             ||  ||||||||||||||||||||||||||||||||||||||||||| ||||||||||||

PS9 Clone  6978  GCGCTACCGATTGTGGAACAAACAGGATTAGACTACGCTTCAAAAAACGAAGGAGCGATG  7037

Strep_263  6958  CATGCGTGTGGACATGATTTCCATACAGCAAGCCTACTTGGAGCCGTTCAAGTGTTGAAG  7017

             ||||| || ||||||||||||||||| || ||| | || |||||||||||||| || |||

PS9 Clone  7038  CATGCATGCGGACATGATTTCCATACCGCTAGCTTGCTGGGAGCCGTTCAAGTATTAAAG  7097

Strep_263  7018  GAGCAAGAAGCTCACTTACAAGGAAAAGTTCGTTTTATTTTCCAACCGGCCGAAGAAAGT  7077

             |  |||||||   | |||||||| ||||||||||||||||||||||| ||||||||||||

PS9 Clone  7098  GCTCAAGAAGACAAATTACAAGGGAAAGTTCGTTTTATTTTCCAACCAGCCGAAGAAAGT  7157

Strep_263  7078  AACCAAGGAGCACGCGCATTGATTGCAGAAGGCGTGCTTGACGGAGTGGACGCGATTATT  7137

             ||||  |||||||||||||||||| | ||||| |||||||| |||||||| |||||||||

PS9 Clone  7158  AACCGTGGAGCACGCGCATTGATTTCTGAAGGTGTGCTTGATGGAGTGGATGCGATTATT  7217

Strep_263  7138  GGATTCCATAATAAACCAGAAATTCCAGTTGGAACGATTGGTGTGAAGGAAGGACCTCTG  7197

             |||||||||||||| |||||| ||||||||||||||||||||||||||||||||||||||

PS9 Clone  7218  GGATTCCATAATAAGCCAGAACTTCCAGTTGGAACGATTGGTGTGAAGGAAGGACCTCTG  7277

Strep_263  7198  ATGGCAGCAGTCGGCCAGTTCAAAGCCGAAATTACAGGAGTAGGAACGCATGCGGCAGCA  7257

             |||||||| ||||||||||| ||||||||||||||||| ||||||||||| || ||||||

PS9 Clone  7278  ATGGCAGCGGTCGGCCAGTTTAAAGCCGAAATTACAGGGGTAGGAACGCACGCTGCAGCA  7337

Strep_263  7258  CCACACAATGGGAATGACCCAATCGTGACGGCCTGCCAAGTGATTGCTAATGCTCAAGCC  7317

             || || || || || ||||| ||||| ||||| |||||||||||||| |||||||||||

PS9 Clone  7338  CCGCATAACGGAAACGACCCCATCGTTACGGCTTGCCAAGTGATTGCAAATGCTCAAGCG  7397

Strep_263  7318  ATCGTTGCTCGTCATACTTCTCCGCTCGAACCAGTGGTATTAAGCGTATCGCATATCGAA  7377

             || ||||| |||||||| || || || |||||||||||||||||||||||||||||||||

PS9 Clone  7398  ATTGTTGCACGTCATACATCCCCACTTGAACCAGTGGTATTAAGCGTATCGCATATCGAA  7457

Strep_263  7378  GCAGGGAATACATGGAATGTAATTCCAGAGAAAGTATTTTTTGAAGGAACGATTCGTACG  7437

             |||||||||||||||||||| |||||||||||||||||||||||||||||||||||||||

PS9 Clone  7458  GCAGGGAATACATGGAATGTGATTCCAGAGAAAGTATTTTTTGAAGGAACGATTCGTACG  7517

Strep_263  7438  TTTAATAAAGACGTGGAACGTAAAATGACCGAGCAATTCGAGAAGATGATTGTCCAAACC  7497

             ||||||||||| ||||||||| |||||||  |||||||||| ||||||||||| |||||

PS9 Clone  7518  TTTAATAAAGAAGTGGAACGTCAAATGACGCAGCAATTCGAAAAGATGATTGTTCAAACG  7577

Strep_263  7498  GCAGATGTTTACGGACAAAAAGGAAGTATCGAATGGATTTTAACGCCACCTGTCGTGCAT  7557

             || ||||||||||| |||||||||||||||||||||||||||||||| || || ||||||

PS9 Clone  7578  GCTGATGTTTACGGGCAAAAAGGAAGTATCGAATGGATTTTAACGCCGCCAGTTGTGCAT  7637

Strep_263  7558  AATGATGCTGCAGTCACAAAAGTGGTTAAAAGTGTGACGGAGAAATTTGCGACAGTGGTG  7617

             ||||| |  | | | |||||||| || | | |    ||||||||||||||||||||||||

PS9 Clone  7638  AATGACGTAGAAATTACAAAAGTAGTGAGACGCACAACGGAGAAATTTGCGACAGTGGTG  7697

Strep_263  7618  ACACCTGAAGTAACTTTAGGAGCAGAAGATTTCGCCAATTATATGGAACACGTTCCAGGA  7677

             ||||||||||| ||||||||||||||||||||||||||||||||||||||||||||||||

PS9 Clone  7698  ACACCTGAAGTGACTTTAGGAGCAGAAGATTTCGCCAATTATATGGAACACGTTCCAGGA  7757

Strep_263  7678  TGTTT  7682

              |||||

PS9 Clone  7758  TGTTT  7762

(c)

>PS9_Oral_Metagenomic_Insert

ACTCGTCATTATTGACGCATATATGATTGTAAAATCAAGAAAAGCAAAGCGAAATTTATAAAGTGTGAAAAGGAGTGAAAACTTTATTTTTTCTAATTAAAATAGCGGAACAATTAGAATTGGAAGATCCGTTTACAGGAAAATATAAATAGGTGCTCGTCGCAGGCCACACGAGCGCCTTGAGGCGTCGCTCTGAGATCAGAGCTATGCCCGAAATTGAAAAGCCACCCGCTAAGCCACTGGATATTTATTATGGGAGAAATTATCATATATGATAATTTCGCAAAGTGTGCTTCATCGATTTGGTTCGCCTTGAGGGTCCATGGTATAATGAATAAGATAAAATAAGAAATGGGGAAACCACATGAAACCATTTGATTACATCGTTGTGGGTGCTGGATTATTCGGTGCGACCTTTGCGCATGAAGCAGCGACACGCGGTTATAAAGTAAAAGTGATTGAGAAGAGAAATCATATTGCGGGGAATATTTATACAAAAGAAGTGGAAGGCATTCAAGTGCACGAATACGGTGCGCATATTTTCCACACGAGCGACAAGAAGATTTGGGATTACGTGCATCAGTTTGCGACATTTAATCGCTATACCAATACTCCAGTTGCCAACTTCAACGGAGAAATCTACAACTTGCCCTTTAACATGAATACTTTCAATAAATTATGGGGTGTGGTGACGCCACAAGAAGCCGAAGCGAAAATTGCTGAACAGCGTGCGGTACTCGGGGACAAAGAGCCTGAAAACTTAGTAGAGCAGGCGATTTCTCTTGTTGGGGAAGATATTTACTACAAGCTCATCAAGGGCTATACGGAGAAACAATGGGGACGTTCGGCTACAGAATTACCAGCATTTATCATTCGCCGCTTGCCGGTTCGTTACACTTACGATAACAACTATTTTAACGATACGTACCAAGGGATTCCGATTGGTGGCTATACGAAGATGATTGAAGCCATGCTGGACCATGAGAATATCGAAGTCGAGTTGAATGTTGATTTCTTTGCGAAAAAAGATGAGTACTTAAGTAGTGGTGCCAAAATCGTCTTCACGGGAATGATTGATGAGTTCTTCGATTATGAACTAGGCACATTAGAATACCGTTCGCTCCGTTTTGAAACAGAAGTCGTGGACGTGGAGAACTATCAAGGAAATGCGGTAGTAAACTATACAGACCGCGAAACGCCATACACTCGTATCATCGAGCATAAGCATTTCGAGTTTGGCACGCAGCCCAAAACGGTGATTACGCGTGAGTATCCTGCCGATTGGAAAGTGGGAGACGAGCCTTATTATCCAGTGAATAATAAAGTCAACAACGACTTGTACGCGCAGTATAAGAAATTAGCGCAGACGGTTCCGCAAGTGATTTTCGGAGGACGCCTTGGACAGTATCGTTATTACGATATGCATCAAGTCATCGCCGCAGCACTAGAAACTGTCGATAAAGAATTCCGTTAAACGCAAGAACACCATATTTGGTCATGGCCAAATATGGTGTTTTTCGATTCTACAGGAGAGATAAAAGTAGTCGGTGTAAGAAATGCAATGAAGCTTAATAGAGAAAAACGTGTGTAGTTTATAAATAGTGAAACCGAAGAATCTTGCGGATTTCATAACCACAATAATGGGGAATGGTATTAAATGCGAAAAAAGCTGGAGTTAGTAATTTATTCCTTACTCCAGCTTTGAGGCTTTAAAGGTGAGAGACTATAGGCTCGAACCGGTACCCCATTATTGTAGTTATGATAAAATCCGCTAGGATTCGTAGGTATCACTAATGATAACTTTTAGATACGTTTTTCTCTATTAAGCATTCACTGTATCTAGTTCTTCGCCGATAGCTTGAAGACGAGAGACAACCTCTTCAAGAGATAGGTTGTGTTCTTTCACATAGCGGTTGCGTGGGTGAACACGACATTCATGGCTGCAACCACGTAAGTATTTGTCTTCGTTCTCTTCTGAAGCTAAGATACGACGGTTACATTCTGGGTTTCCACAGTTTACGTAACGTTCGCAAGGTGTACCATCGAACCAGTCTTTCCCAATTACTACTGGATCTACGTGGTTGATGTCAACAGCGATACGTTCGTCAAATACGTACATTTTTCCATCCCATAATTCGCCTTGAACTTCTGGGTCTTTCCCGTAAGTTGCGATACCGCCGTGTAATTGACCGACATCTTTGTAGCCTTCACGAACCATCCAGCCAGAGAATTTCTCACAG

CGAACGCCACCTGTACAGTAAACAACGACACGTTTGTCCATGAATTTCTCTTTGTTATCGCGAACCCATTGTGGTAATTCACGGAAGTTGCGAATGTCAGGACGGATGGCACCGCGGAAGTGACCTAGGTCGTACTCGTAGTCGTTACGAGTGTCTAGTACAACAGTGTTTTCGTCAAGTAGGGCTTCTTTAAACTCTTGTGGAGAAAGGTAAGCCCCTGTTGTTTCAAGTGGGTTAATATCGTTGTCGAAGTCGTTATCTTCTAAACCAAGGTGTACGATTTCTTTTTTGTAACGAACGAACATCTTCTTGAAGGCTTGCTCGCTTTCTTCGTCGATTTTAAACCATAAGTCTTCCATCCCTGGAAGAGAGTGAACATAGTCCATATATTTTTGAGTTGTTTCGTAATCACCAGATACCGTTCCGTTAATTCCTTCGTCAGCGACAAGGATACGTCCTTTTAATCCGATTGATTTGCAGAATGCTAAATGATCTGCCGCGAATTGTTCCGCATTTTCGATTGGAACATATTTATAGTAAAGTAAAACACGAATGTCTTTTGCCATATTGTAAATCTTTCATCCTTTTCTAATTTGTAATGATTCTATTGTAACTGTATTGAGAATGATTATCAATGAATTCGATTGTGAAAAAGTTCGAAAGGTATTGACATGACGGTATTTTACGGTATAATTCAGATATAACGTTAAATTTATCAGATTGGAGGAGAAATCAATGAACAACTTAAACGTAAAACAAGTACAAGATTTGAGAAACCTTCCAATTTTGGTGAATGAAGTATAGCGCATTTTTATAGATAAGTTTAAGTGAGCCGTTACTTTTTCCATGCGTGGGGAAGGTGACGGCTTTTTTGATGGAGGAAAACGATGAACGATTTATTAAAAGTCATTATTAATTTTATAAAGAAACATCCGATGCGCTACCTTGTTAGTTTTATTTTGATGATCGGAAGTAGTATTGCGGCGGTGTACCCAGCGCGTATTATCGGACAAGTTGTTGATAAAATCGTAGCGAGCGAACTGAATGCCGAGTGGCTTGGGACACAACTCGTGATTTTAGTCGGGATTATTCTTGTGGCGTATATTACGGAGAGTATTTGGACATATTTTATTTTTATTGGGTATTATGAAATTCAAAAAGAATTACGTGTGAAGTTACTACGTAATAATTTACGGAAGAAAATTCCGTTTTATGCGCATTTTAGAACGGGCGAAATTATTACGCGTAGCAGTGAAGACGTTACAACGATTGGCGATATGATGGGGTTTGGGATGTTTGCATTGATGAACTCTACATTGCTGATGAGCGTATCGATTTATATGATGGTCACAACGATTTCATTGCCACTGACCATCGCAGCGATTTTGCCACTGCCAATCCTTTCGTATCTTGTATATAAATGGGGATTCGATTTAGAAGAAGAGTACAACAAGGCGCAAAATGCAGTTTCACAATTAAATAATGAAGTGCTTGAGATGATTGACGGGACGTATGTGATTCGTGCTTACGGGCAAGAAGATGCGATGATGGATGAGTTCAGGGCGAAAACGAAAAAGGCCATGAAACAAAATATTATCGTGACTGAAATTGAATCGCGCTTTATTCCACTGGCGCAATTATTTATGATGATTAGCTTTACCATTGCCCTTTTCTACGGTGGGTATCTAGTATCGACTGGGGCTATTCTAGTCGGGGATGTCATTGCCTTCCAAGTCTATATGGGGGCGATTATGTGGCCGATGTTTATGATTGGCGATATTATTACGAACTATAAACGCGGAAAAGTGGCGACGGAGCGTATTAATGAAGTGTTGAAACATGACGATGAAATTGAACGCGGCGGTACAAAAACGCTCGAGACGATTGAATCCATTGAGTTTAAGGACTTCCATTTTATGTATCCAGGCGAAGAGGCACCATTATTAAAAGAGATTAACCTTACGTTACGTAAAGGCGAGACGCTTGGAATCGTTGGAAAAACGGGTTCTGGGAAGACGACGCTCTTGATGCAATTATTACATCAATTTCCGTACCGAGGAGAGAAGCTGCTCATTAACGGAGAGCCATTGATTGATTACGACACTCAATCGGTGGCAGGGCATCTAGCCTATGTGCCACAAGAACACACCCTTTTCTCACGCACGATTCGCGAGAATATGTTATTCGGAAAAGAGGATGCAACGGATGATGAAATTTGGGAAGCGTTGACGCTAGCCTCTTTTGAAGGAGACGTGAAACGAATGCCAGACGAGCTCGATACGATGGTCGGAGAAAAAGGGGTATCGCTCAGTGGAGGTCAAAAACAACGCTTATCGATTGCTCGTGCTTTCTTACGCAACCGTGAATGCTTAATTTTGGATGATGCGTTATCTGCAGTTGATGCGAAAACGGAAAGGGAAATTATCTCGCACTTGCAACAAGAACGCGGAGGTTGTATGAATATCATTTCTGCGCACAGACTTTCTGCAATTCGTCATGCGGATGAAATTATTGTGATGAATGAAGGACGTATTAGTGAGAGGGGTACCCACGAGGAGCTGCTCGAACAACGAGGATGGTACTATGAACAGTATCTCACACAAGAAATGGAGGAGGAAATCGAATGAGAACGATGAAGCGATTATTAAGCTACCTCCGTTATGAGAAAAAAGGAGTTCTGATTGGACTCTTCTGCCTGTTACTCTCAACAGGAGCAACC

TTAACAGGGCCACTCGTTGCGAAACACATTATCGATAATGTGATTACTCCGATGGGGCAGGCGCATGATTTCAAAGCAGGTGGCCTACTTTTATGGGTTGGTATTTACGTGACAGTGAACTTAGTGGGAGTTGCTGGGGCCTATTTAAACCGTGTATATATGAAGACTCTCTCAAACCGTATTGCAAAACGCATTCGTGACGAAGTGTTCGAGCATGTACAAACCTTGCCGGTATCGTACTTCGACCATTTACCAGCTGGGAAAGTGGTTTCTAGAATTACAAGTGATACAGAATCAGTGCGTGCAAACTTCTACGTGAGTGGGATTTCGACGCTTTTCAGTACGATTGTGATGTTGGTAGGTGTTTATATAACCATTTTCTTATTAAACGCAACGCTAGGACTCGTATTATTATTCCTAGTTCCTGTGATGATTCTATGGCAGAGAACCGTTGCTACGAAGCAGAAAAAATACTATTCCGAAAATCGTGAACTCTATAGTCAGTTGAGTGGACAATTAAACGAAAGCATTCAAGGAGCAGGCATCGTTCAAGCCTTCCAGCAAGAAGAAAAAATTGTTGCAGAATATGATGCCACGGCAACTTCTTGGGTAGAAGTTGGTCGTAAGGAATTAATTCTTGAGTCGTACTTCTCGTGGAGTCTTGTCGGCATGCTTCGAAACATTACTCATTTTGGAGTTATCTATTATTTCAGTATGCAGTTTATCGGTGGAACACTCGGGATTTCAGCAGGTCTTTTATATGCATTTATTGACTACATTAATCGTATATATGAGCCGATTCAAACTTTTATGAATGTCGTGTCTGGCTTCCAGCAATCAATGGCTGCTGGTGACCGTGTGTTTGAACTAATGGATACACCGAGCGAGGAATCGGGAGAAGAGCTCTTCACGTTTGATGAAGGATGTATCGAATTTAAAGATGTGAGTTTTGAGTACACAGCGGGAGTTCCTGTGTTGAAACACTTGAATTTCACGGTAGAACCAGGACAGACGGTTGCTTTTGTCGGACATACTGGTTCAGGGAAATCATCCATTATGAACTTACTGTTTCGCTTCTACGACCCAACGAGTGGTGCTATCTTCATCGATGGCAAAAACACACGCGACTTTAACAGACGTAGTGTTCGAAGCGAGATGGGAATCGTTCTTCAAGATCCGTACCTCTTTACAGGAACGATTGCTTCAAACGTAGGGCTCAACAATGAATCGATTGAGCCTGAGACGATAAAAGAGGCGATTATTAAAGTGGGTGGAGGACATCTACTTACGAAGAGTGACAAGGGCTTGGACTACGAGGTGAAAGAAAAAGGAATGGATTTCTCTTCAGGAGAACGCCAACTGATTTCATTTGCTCGTGCGATCGTCTTTGACCCGAAAATCTTAATTTTAGACGAGGCAACTTCGCATATCGATACCGAGACAGAAGAGATTATTCAGAATGCAATTAATGTCGTCAAAGAAGGGCGTACAACCTTTATGATTGCTCACCGACTTTCCACAATTGCTCATGCTGACCAAATTTTTGTGTTGGATAAAGGAGAAATTGTAGAACGTGGAACGCATGATGAATTGCTTCAACTGCAAGGTCAATATGCCGAAATGGTCGCCCTACAAAAAGGATAATTTTAAAAAATACAAGACGCTGTTTCCTTTGAAACAGTGTCTTTTCTTTCGTAGCGGTAAACTTTATTTTTTTAGTGTTTTTTCAAGGGAATTCATGGTGCATTACGCCTCTTAGTTTGTTACAATGTGTAGGAAACTGATTGAGGAGAGAACGTATGATTACGAAAGAAGTTATTGAACGTGCAACAACGCACCGCCGCCATTTACATATGTATCCAGAAGTATCTGGAGAAGAAGTTGAAACAACGCGCTATATCCGAGAAGCCCTAGAAGCGATGGGACTTACTTGCTGGAATTTAAAGTCTAAGACGGGAGTGGTTGCAGAACTCGGAAACGGTGAGGGACCAACATTAGCCTTACGTGCCGACATCGATGCGCTACCGATTGTGGAACAAACAGGATTAGACTACGCTTCAAAAAACGAAGGAGCGATGCATGCATGCGGACATGATTTCCATACCGCTAGCTTGCTGGGAGCCGTTCAAGTATTAAAGGCTCAAGAAGACAAATTACAAGGGAAAGTTCGTTTTATTTTCCAACCAGCCGAAGAAAGTAACCGTGGAGCACGCGCATTGATTTCTGAAGGTGTGCTTGATGGAGTGGATGCGATTATTGGATTCCATAATAAGCCAGAACTTCCAGTTGGAACGATTGGTGTGAAGGAAGGACCTCTGATGGCAGCGGTCGGCCAGTTTAAAGCCGAAATTACAGGGGTAGGAACGCACGCTGCAGCACCGCATAACGGAAACGACCCCATCGTTACGGCTTGCCAAGTGATTGCAAATGCTCAAGCGATTGTTGCACGTCATACATCCCCACTTGAACCAGTGGTATTAAGCGTATCGCATATCGAAGCAGGGAATACATGGAATGTGATTCCAGAGAAAGTATTTTTTGAAGGAACGATTCGTACGTTTAATAAAGAAGTGGAACGTCAAATGACGCAGCAATTCGAAAAGATGATTGTTCAAACGGCTGATGTTTACGGGCAAAAAGGAAGTATCGAATGGATTTTAACGCCGCCAGTTGTGCATAATGACGTAGAAATTACAAAAGTAGTGAGACGCACAACGGAGAAATTTGCGACAGTGGTGACACCTGAAGTGACTTTAGGAGCAGAAGATTTCGCCAATTATATGGAACACGTTCCAGGATGTTTTGT

Figure 1. (a) BlastN alignment of the PS9 sequence with *Granulicatella adiacens* ATCC 49175. There was no alignment of the sequences from 1-318 bp in the PS9 sequence. They are related between 319 and 1494 bp and then there is an inversion in the region 1608 – 1709 and then they are similar all the way through to the end of the sequence. HindIII sites in PS9 are highlighted in yellow and the 46 bp region with nucleotide identity to *Lactobacillus buchneri* is underlined (4610-4655). ORFs are present in PS9 are highlighted in blue and their start codons are underlined. (b) BlastN alignment of the PS9 and *Streptococcus*_sp_263_SSPC. The sequences are related from position 1 – 1519, there is then an inversion in the region 1600 – 1789 and then they are similar all the way through to the end of the sequence. HindIII sites in PS9 are highlighted in yellow and the 46 bp region with nucleotide identity to *Lactobacillus buchneri* is underlined (4540-4585). ORFs are present in PS9 are highlighted in blue and their start codons are underlined. (c) The sequenced 7,765 bp PS9 insert.
